# Automated Abdominal Aortic Calcification Scores and Atherosclerotic Cardiovascular Disease in the UK Biobank Imaging Study

**DOI:** 10.1016/j.jacadv.2025.102570

**Published:** 2026-01-29

**Authors:** Marc Sim, James Webster, Cassandra Smith, Afsah Saleem, Syed Zulqarnain Gilani, Carlos J. Toro-Huamanchumo, David Suter, Gemma Figtree, Anne Karine Lagendijk, Emma L. Duncan, Carl Schultz, Pawel Szulc, Joseph Hung, Wai H. Lim, Parminder Raina, Nicola P. Bondonno, Richard Woodman, Jonathan M. Hodgson, Douglas P. Kiel, Richard L. Prince, William D. Leslie, John P. Kemp, Nicholas C. Harvey, John T. Schousboe, Joshua R. Lewis

**Affiliations:** a Nutrition and Health Innovation Research Institute, Edith Cowan University, Perth, Australia; b Medical School, The University of Western Australia, Perth, Australia; c Applied Health Research Unit, Nuffield Department of Population Health, University of Oxford, Oxford, United Kingdom; d Centre for AI and ML, School of Science, Edith Cowan University, Perth, Australia; e Computer Science and Software Engineering, The University of Western Australia, Perth, Australia; f OBEMET Center for Obesity and Metabolic Health, Lima, Peru; g Universidad San Ignacio de Loyola, Lima, Peru; h Faculty of Health and Medicine, The University of Sydney, Sydney, New South Wales, Australia; i Kolling Institute of Medical Research, Sydney, New South Wales, Australia; j Centre for Cell Biology and Chronic Disease, Institute for Molecular Bioscience, The University of Queensland, Brisbane, Queensland, Australia; k School of Biomedical Sciences, Faculty of Medicine, The University of Queensland, Brisbane, Queensland, Australia; l Department of Twin Research and Genetic Epidemiology, School of Life Course and Population Sciences, King’s College London, London, United Kingdom; m Department of Endocrinology, Guy’s and St Thomas’ NHS Foundation Trust, London, United Kingdom; n INSERM UMR 1033, University of Lyon, Hospices Civils de Lyon, Lyon, France; o Cardiovascular Epidemiology Research Centre, The University of Western Australia, Perth, Australia; p Department of Renal Medicine and Transplantation, Sir Charles Gairdner Hospital, Perth, Australia; q McMaster Institute for Research on Aging, McMaster University, Hamilton, Ontario, Canada; r Department of Health Research Methods, Evidence, and Impact, McMaster University, Hamilton, Ontario, Canada; s Labarge Centre for Mobility in Aging, McMaster University, Hamilton, Ontario, Canada; t Danish Cancer Institute, Copenhagen, Denmark; u Flinders Health and Medical Research Institute, College of Medicine and Public Health, Flinders University, South Australia, Australia; v Department of Medicine Beth Israel Deaconess Medical Center and Harvard Medical School, Hinda and Arthur Marcus Institute for Aging Research, Hebrew SeniorLife, Boston, Massachusetts, USA; w Departments of Medicine and Radiology, University of Manitoba, Winnipeg, Canada; x Mater Research Institute, The University of Queensland, Translational Research Institute, Woolloongabba, Queensland, Australia; y MRC Lifecourse Epidemiology Centre, University of Southampton, Southampton, United Kingdom; z NIHR Southampton Biomedical Research Centre, University of Southampton and University Hospital Southampton NHS Foundation Trust, Southampton, United Kingdom; aa Park Nicollet Clinic and HealthPartners Institute, HealthPartners, Minneapolis, Minnesota, USA; ab Division of Health Policy and Management, University of Minnesota, Minneapolis, Minnesota, USA

**Keywords:** atherosclerosis, bone density, cardiovascular disease, dual-energy X-ray absorptiometry, vascular calcification

## Abstract

**Background:**

Abdominal aortic calcification (AAC) is a subclinical measure of atherosclerotic cardiovascular disease (ASCVD). AAC can be captured on lateral spine images obtained from bone density machines during routine osteoporosis screening. Identifying individuals with AAC provides a new opportunity to prevent disease progression.

**Objectives:**

The aim of the study was to externally validate a machine learning-derived AAC 24-point algorithm (ML-AAC24) with incident ASCVD.

**Methods:**

Middle-aged individuals from the UK Biobank Imaging Study with lateral spine images, obtained via dual-energy x-ray absorptiometry, were included. ML-AAC24 scores were grouped as low (<2), moderate (2 to <6), and high (≥6). Linked health records were used to identify ASCVD-associated events, including hospitalizations and death.

**Results:**

Among 53,611 participants (52% female; mean age 65 years), 78.2% had low, 16.4% had moderate, and 5.4% had high ML-AAC24. After excluding people with prevalent ASCVD or missing data, 1,163 (2.3%) of 50,923 people had an incident ASCVD event over a median follow-up of 4.1 [3.0-5.5] years. In age- and sex-adjusted analysis, compared to those with low ML-AAC24, those with moderate (HR: 1.80 [95% CI: 1.57-2.08]) and high ML-AAC24 (HR: 2.87 [95% CI: 2.39-3.44]) had a higher HR for incident ASCVD. Results remained comparable after adjustment for established ASCVD risk factors. Consistent patterns were observed when considering incident coronary artery disease, myocardial infarction, and stroke.

**Conclusions:**

Assessing ML-AAC24 on lateral spine images offers a new and promising screening method to identify people with higher risk of incident ASVD events.

Cardiovascular disease (CVD) is a leading cause of death with an estimated 19.8 million CVD-related deaths worldwide in 2022.^1^ Furthermore, CVD, which is primarily driven by atherosclerotic CVD (ASCVD) is a major contributor to life years lost, reduced quality of life, and excess health system costs.^1^ While major modifiable risk factors (eg, hypertension, smoking, diabetes, and hypercholesterolemia) are important to detect and treat ASCVD, 11% to 27% of patients with a first time myocardial infarction (MI) event have no prior standard modifiable risk factors (SMuRFs).^2^ Furthermore, 40% of cardiovascular events are not explained by SMuRFs.^3^ This highlights the substantial unmet need for improved early detection of subclinical ASCVD.

Abdominal aortic calcification (AAC) is a subclinical measure of ASCVD that is common in middle-aged individuals.^4^^,^^5^ AAC often precedes coronary artery calcification (CAC),^6^ and is strongly related to atherosclerosis in other vascular beds, including the carotid arteries.^7^, ^8^, ^9^ Notably, AAC can be assessed using noninvasive computed tomography machines or lateral spine images (LSIs) obtained using dual-energy X-ray absorptiometry (DXA) and X-rays. These LSIs are used primarily to identify vertebral fractures and osteoarthritis.^10^ DXA-derived LSIs can be obtained at a fraction of the cost and radiation exposure of computed tomography or X-rays, making them an attractive proposition for widespread screening. AAC is typically scored on a semiquantitative 24-point scale (AAC24). Regardless of imaging modality, major limitations of AAC assessment include its time-consuming nature and the lack of expert readers.^10^

Several cohort studies with smaller numbers of annotated images have tried to automate AAC24 scoring with limited success for identifying those at greater CVD risk.^11^^,^^12^ One such study undertaken in the UK Biobank reported modest correlations (r = 0.67) compared to human annotations.^11^ Other key issues identified included: 1) little information on the expertise or training of annotators, including large inter-reader variability; 2) relatively modest associations with MI with no evidence of a gradient of risk (dose-response); and 3) lack of adjustment for traditional CVD risk factors. Alternatively, our recently published machine learning algorithm for AAC24 scoring (ML-AAC24) was trained and tested on a large dataset of 5012 DXA-derived LSI with human expert annotations. A high level of agreement (intraclass correlation coefficients [ICCs] all >0.8) was also recorded between predicted and expert scores across multiple cohorts.^13^ Our preliminary external validation demonstrated that ML-AAC24 extent was strongly associated with incident CVD in 8,565 older individuals (93% female, mean age ∼76 years) undergoing routine osteoporosis screening.^13^

To build on our previous work,^13^ we sought to externally validate our ML-AAC24 algorithm against expert assessment and investigate its association with incident ASCVD in middle-aged to older adults in the UK Biobank Imaging Study. Furthermore, we examined whether associations were independent of standard nonmodifiable and modifiable CVD risk factors.

## Methods

### Participants

Participants from the UK Biobank Imaging Study with a DXA-derived LSI were included (Supplemental Figure 1: prospective cohort with incident clinical outcomes). Our ML-AAC24 algorithm was applied to LSIs from the first imaging visit (instance 2) for each participant (2014+) (n = 53,611). For the prospective cohort, only individuals with LSIs obtained between study commencement (2014) and end of follow-up were included. This is detailed in the clinical outcomes section. After excluding individuals with: 1) no follow-up (eg, LSI obtained after region-specific end of follow-up for linked health records [n = 435]; 2) known prevalent ASCVD (n = 2,237); or 3) loss to follow-up or withdrawals (n = 16), 50,923 adults were eligible for the primary survival analysis adjusted for age and sex (model 1). When the model was adjusted for various covariates, sample size varied between 47,148 (model 2) and 41,175 (model 3).

### Machine learning-derived abdominal aortic calcification 24 scores (ML-AAC24)

DXA-derived deidentified LSIs were captured using an iDXA bone density machine (GE Healthcare). The LSIs were converted to 8-bit DICOM format and made available to researchers through the UK Biobank Imaging Study. The initial ML-AAC24 algorithm was developed using 5012 DXA-derived LSIs from four independent cohorts.^13^ This algorithm demonstrated substantial agreement with expert-assessed AAC24 scores and showed a strong association with CVD events (eg, adjusted HR >2 for high-risk vs low-risk groups) from images with no expert scores.^13^ To adapt the ML-AAC24 algorithm for the UK Biobank cohort, we paired 497 randomly selected LSIs with expert annotations (provided by J.T.S.). These images and expert scores were then input into the pretrained ML-AAC24 model for the fine-tuning of model parameters. As detailed in the Supplemental Text, our ML-AAC24 algorithm consists of 3 steps: 1) image preprocessing to extract region of interest; 2) feature extraction using fine-tuning; and 3) AAC24 score prediction using regression. The ML-AAC24 scores for the remaining LSIs in the cohort were then predicted by passing only the scans through the fine-tuned algorithm. The extent of ML-AAC24 was categorized using established categories: low (ML-AAC24 <2), moderate (ML-AAC24 2 to ≤5), or high (ML-AAC24 ≥6). These thresholds have been validated against CVD when considering both ML-AAC24^13^^,^^14^ and expert-assessed AAC24.^15^

### Clinical outcomes

The primary outcome was the first ASCVD event as previously defined^16^ from International Classification of Diseases-9 and International Classification of Diseases-10 codes comprising: acute MI, ischemic stroke, or their acute complications, in any diagnosis position in hospital records; coronary revascularization procedures (coronary artery bypass graft surgery or percutaneous angioplasty/stent placement, Office of Population Censuses and Surveys-4 [OPCS] codes); or death register indicating MI or ischemic stroke as a cause of death (whether a main or contributory cause). Incident coronary artery disease (CAD), MI, and ischemic stroke were also considered as separate secondary outcomes.^16^ Of note, high accuracy regarding the use of these data to identify first stroke (positive predictive value 89% 95% CI 82-94%)^17^ and MI (positive predictive value approximately 75%-100%) has been reported.^18^ Date of first DXA-derived LSI for each participant was considered the index date (baseline). End of follow-up censoring dates differed due to the availability of linked health records based on region (England: 31 Oct 2022, Wales: 31 May 2022, Scotland: 31 August 2022), as provided by the UK Biobank at time of data extraction (November 2023). Any ASCVD event during this time was considered an incident, while any ASCVD event prior to the index date was considered prevalent. Clinical outcomes are detailed in Supplemental Text and Supplemental Table 1.

### Covariates

Age at imaging was calculated using LSI date and birth year. Physiological and lifestyle factors included sex (male/female), self-reported ethnicity (White/Black/Asian/Other), height and weight (from stadiometer and digital scales), body mass index (BMI), systolic blood pressure (SBP), high-density lipoprotein (HDL), total cholesterol, prevalent diabetes, self-reported smoking status (never/current/previous), and physical activity (low/moderate/high) using the short International Physical Activity Questionnaire.^19^ Country of residence (England/Wales/Scotland) and year of imaging were also considered. Detailed covariate descriptions are provided in Supplemental Text and Supplemental Table 1.

### Statistical analysis

Statistical analyses were performed in Stata/MP (version 18.0). Pearson’s correlations, ICC, and Bland-Altman plots (created using Pyton V3.9.16) were used to examine the relationship between ML-AAC24 and human-assessed AAC24 in the 497 images with expert annotations. For the prospective analysis, cumulative incidence curves and Cox proportional-hazards analyses were used to examine associations between the extent of ML-AAC24 (exposure) and incident ASCVD (primary outcome), including CAD, MI, and stroke (secondary outcomes), with separate analyses by sex. Gradient of ASCVD risk was explored using cumulative incidence curves, comparing deciles 8, 9, and 10 of ML-AAC24 to a reference group comprising of deciles 1 to 7 combined, representing no calcification (all ML-AAC24 <1). Proportional hazards assumptions were inspected graphically using Schoenfeld residuals (no violations detected). For all analyses, we considered 3 models of adjustment.

• Model 1: age and sex;
• Model 2: Model 1 + BMI, ethnicity, SBP, physical activity, smoking, prevalent diabetes, country of residence, and year the LSI was obtained;
• Model 3: Model 2, excluding BMI, but including total cholesterol and HDL.

Models were based on key risk factors included in the Framingham Risk Score.^20^ Specifically, the Framingham Risk Score proposes a simpler model using BMI when lipids are unavailable (eg, model 2) or an expanded model including lipids such as cholesterol and HDL instead of BMI (eg, model 3).^20^ For each model, only people with all covariates were included; consequently, sample sizes differed. Supplemental Figure 2 displays a directed acyclic graph depicting the hypothesized relationship between ML-AAC24 and incident ASCVD, with the confounders adjusted for in model 3. The log-likelihood chi-square (chi-square) statistic was also used to evaluate the significance of removing individual predictor variables from model 3 when considering the relationship between ML-AAC24 extent and ASCVD. The resulting change in log-likelihood chi-square for each predictor variable was used for ranking its relative importance in relation to ASCVD. A larger change in log-likelihood chi-square upon the removal of a variable from the final model indicates a variable of greater importance. To determine whether adding extent of ML-AAC24 to standard risk factors (model 3) improved model discrimination for ASCVD, receiver operating characteristic, and category-free net reclassification index analysis was undertaken. Statistical significance was set at a two-sided type one error rate of *P* < 0.05 for all tests.

### Sensitivity analysis

Due to their effect on lipids and blood pressure, cholesterol and blood pressure medications may be viewed as a confounder or mediator^21^ when considering the relationship between ML-AAC24 and ASCVD. As such, rather than include medication use in the primary analysis, we undertook sensitivity analysis where these were included as additional covariates to model 3. We examined the presence of any ML-AAC24 (yes ≥1, no <1) with incident ASCVD, including its components. To remove potential subset bias between models due to covariate availability for the primary analysis between extent of ML-AAC24 and incident ASCVD, the analysis for models 1 and 2 was restricted to the 41,175 participants in model 3.

We also undertook exploratory cross-sectional analysis in a subcohort (termed the imaging cohort) (Supplemental Figure 3), where we examined the relationship between ML-AAC24 and another measure of atherosclerosis, carotid intima media thickness (CIMT) (described in Supplemental Text).^22^ All CIMT measures were obtained at the same time point as DXA. Participants missing any CIMT measures were excluded (Supplemental Table 1). We also examined the relationship between ML-AAC24 extent and prevalent ASCVD. Finally, to determine if the relationship between extent of both ML-AAC24 and CIMT with incident ASCVD were independent of each other, further analysis simultaneously including both these measures were also performed. Detailed descriptions of all exploratory analysis relating to ML-AAC24, CIMT, prevalent and incident ASCVD are described in the Supplementary sensitivity analysis.

### Ethics

UK Biobank ethical approval was obtained from the Northwest Multicenter Research Ethics Committee UK (REC reference: 11/NW/03/820). All participants provided written informed consent. This project was approved by the UK Biobank (Application #93712) and conforms to STROBE guidelines.^23^

## Results

### ML-AAC24 scores

The pretrained fine-tuned algorithm demonstrated a Pearson’s correlation of r > 0.83, 95% CI: 0.80-0.86) (Supplemental Figure 4A) and good agreement (ICC 0.82, 95% CI: 0.79-0.85) with expert scores. The three-group accuracy (low, moderate, and high) was 88% (area under the curve [AUC] 0.77, 95% CI: 0.73-0.81, positive predictive value: 80%; negative predictive value: 90%) (Supplemental Table 2), with Bland-Altman plots indicating a good overall agreement with no clear sign of bias (Supplemental Figure 4D). However, the ML-AAC24 algorithm tended to underestimate AAC24 scores compared to expert assessment. The confusion matrix also indicated no low ML-AAC24 cases were misclassified as high, and no high ML-AAC24 cases were misclassified as low (Supplemental Figure 4B). The algorithm was then used to predict ML-AAC24 scores with the distribution presented in Supplemental Figure 4C. Examples of qualitative ML-AAC24 scoring performance with GradCams activation maps are provided in Supplemental Figure 5. Baseline characteristics of the 53,611 participants are described in Table 1. Of these participants, 78.2%, 16.4%, and 5.4% had low, moderate, or high ML-AAC24, respectively.

**Table 1:** Baseline Characteristics of Participants

Characteristic	All Participants (N = 53,611)	Low ML-AAC24 (n = 41,934, 78.2%)	Moderate ML-AAC24 (n = 8,778, 16.4%)	High ML-AAC24 (n = 2,899, 5.4%)
Age at imaging (y)	64.7 ± 7.8	63.2 ± 7.5	69.4 ± 6.3	72.3 ± 5.3
Sex				
Female, n (%)	27,689 (51.7)	22,573 (53.8)	3,979 (45.3)	1,137 (39.2)
Male, n (%)	25,921 (48.4)	19,361 (46.2)	4,799 (54.7)	1761 (60.8)
Body mass index (kg/m^2^)	26.6 ± 4.4	26.6 ± 4.5	26.4 ± 4.1	27.0 ± 4.0
Ethnicity, n (%)^a^				
White	53,489 (96.8)	40,350 (96.5)	8,573 (97.9)	2,850 (98.5)
Black	372 (0.7)	353 (0.8)	19 (0.2)	0 (0.0)
Asian	785 (1.5)	640 (1.5)	106 (1.2)	39 (1.4)
Other	559 (1.1)	494 (1.2)	59 (0.7)	6 (0.2)
IPAQ group, n (%)^b^				
Low	6,238 (12.3)	4,902 (12.4)	983 (12.0)	353 (13.0)
Moderate	21,158 (41.8)	16,625 (41.9)	3,369 (41.1)	1,164 (43.0)
High	23,220 (45.9)	18,180 (45.8)	3,849 (47.0)	1,191 (44.0)
Smoking status, n (%)^c^				
Never	33,229 (62.0)	27,641 (65.9)	4,447 (50.7)	1,141 (39.4)
Former	18,470 (34.5)	12,971 (30.9)	3,902 (44.5)	1,597 (55.1)
Current	1893 (3.5)	1,311 (3.1)	423 (4.8)	159 (5.5)
Prevalent diabetes, yes (%)	1776 (3.3)	1,016 (2.4)	449 (5.2)	311 (10.7)
Prevalent ASCVD, n (%)	2,249 (4.2)	1,113 (2.7)	661 (7.5)	475 (16.4)
Region, n (%)^d^				
England	50,642 (94.5)	39,635 (94.5)	8,265 (94.2)	2,742 (94.6)
Scotland	2,577 (4.8)	2002 (4.8)	438 (5.0)	137 (4.7)
Wales	388 (0.7)	294 (0.7)	75 (0.9)	19 (0.7)
Year of imaging, n (%)^e^				
2014	1,847 (3.6)	1,492 (3.6)	279 (3.2)	76 (2.6)
2015	5,129 (9.6)	4,614 (9.9)	742 (8.5)	223 (7.7)
2016	5,175 (9.7)	4,199 (10.0)	743 (8.5)	233 (8.0)
2017	7,596 (14.2)	6,099 (14.6)	1,149 (13.1)	348 (12.0)
2018	10,560 (19.7)	8,371 (20.0)	1,654 (18.8)	535 (18.5)
2019	11,922 (22.4)	9,260 (22.1)	1,998 (22.8)	664 (22.9)
2020	1882 (3.5)	1,273 (3.0)	426 (4.9)	183 (6.3)
2021	1,463 (2.7)	978 (2.3)	335 (3.8)	150 (5.2)
2022	8,025 (15.0)	6,088 (14.5)	1,452 (16.5)	485 (16.8)

Values are mean ± SD or n (%). Baseline characteristics in UK Biobank imaging study participants by extent of machine learning-derived abdominal aortic calcification scores (ML-AAC24).

ASCVD = atherosclerotic cardiovascular disease; IPAQ = international physical activity questionnaire; ML-AAC24 = machine learning-derived abdominal aortic calcification 24-point algorithm.

a n = 53,489.

b n = 50,616.

c n = 53,592.

d n = 53,607.

e n = 53,599.

### ML-AAC24 with incident ASCVD events

**Figure 1:**
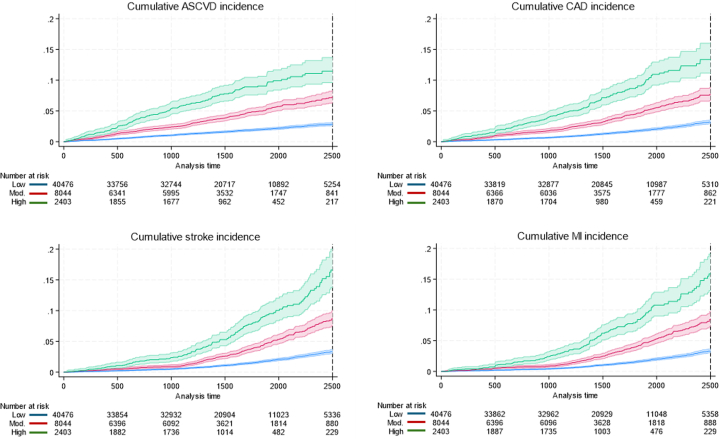
Machine Learning-Derived Abdominal Aortic Calcification 24-Point Algorithm Extent With Atherosclerotic Cardiovascular Disease Cumulative incidence curves for the relationship between machine learning-derived abdominal aortic calcification by severity with ASCVD, CAD, MI, and stroke. Long-rank test *P* < 0.001 for all analyses. X-axis was truncated to 2,500 days for visual representation purposes due to low number of individuals with follow-up beyond that time. ASCVD = atherosclerotic cardiovascular disease; CAD = coronary artery disease; MI = myocardial infarction.

Over a median [IQR] follow-up of 4.1 [3.0-5.5] years (207,224 total person-years, model 1), 1,163 (2.3%) of the 50,923 participants experienced an ASCVD event (Table 2). Individuals with low ML-AAC24 had lower rates of ASCVD events (1.7%), compared to the moderate (3.9%) and high ML-AAC24 groups (6.8%) (*P* for trend <0.001) (Figure 1). In age- and sex-adjusted analyses, participants with moderate (HR: 1.80, 95% CI: 1.57-2.08) and high (HR: 2.87, 95% CI: 2.39-3.44) ML-AAC24 had greater hazards for ASCVD events, compared to low ML-AAC24. Similar results were observed in the standard risk factor-adjusted analysis (model 3; moderate: HR: 1.66, 95% CI: 1.42-1.95; high: HR: 2.46, 95% CI: 2.00-3.02) (Table 2). Results were comparable when stratified by sex (Supplemental Tables 3 and 4) or when incorporating CVD medications into model 3 (Supplemental Table 5). When adding ML-AAC24 extent (low, moderate, and high) to model 1 (plus region and year of imaging), discrimination (C-statistic) of those who subsequently experienced an ASCVD event modestly improved by 0.013 (*P* < 0.001) from 0.750 (95% CI: 0.736-0.764) to 0.763 (95% CI: 0.749-0.777). Similarly, when adding traditional CVD risk factors to model 1, the discrimination improved by 0.017 to 0.767 (95% CI: 0.753-0.781). When ML-AAC24 was added to model 3, the AUC increased by 0.009 to 0.776 (95% CI: 0.763-0.790; *P* < 0.001). The net reclassification index was 0.371 (*P* < 0.001). This means the updated model more appropriately shifted risk categories. Specifically, 7.8% of participants who developed ASCVD were incorrectly moved to lower risk, whilst 44.9% of participants who remained event-free were correctly moved to lower risk.

**Table 2:** Machine Learning-Assessed Abdominal Aortic Calcification Extent and Atherosclerotic Cardiovascular Disease

Outcomes			Low ML-AAC24 HR (95% CI)	Moderate ML-AAC24 HR (95% CI)	High ML-AAC24 HR (95% CI)
Atherosclerotic cardiovascular disease		Events, n (%)^a^	684 (1.7)	315 (3.9)	164 (6.8)
	Model 1^b^	1,163 (2.3%)	Ref 1.0	1.80 (1.57-2.08)^e^	2.87 (2.39-3.44)^e^
	Model 2^c^	1,089 (2.3%)	Ref 1.0	1.76 (1.52-2.04)^e^	2.52 (2.08-3.06)^e^
	Model 3^d^	933 (2.3%)	Ref 1.0	1.66 (1.42-1.95)^e^	2.46 (2.00-3.02)^e^
Coronary artery disease		Events, n (%)^a^	394 (1.0)	209 (2.6)	111 (4.6)
	Model 1^b^	714 (1.4%)	Ref 1.0	2.14 (1.79-2.56)^e^	3.48 (2.77-4.37)^e^
	Model 2^c^	667 (1.4%)	Ref 1.0	2.08 (1.72-2.50)^e^	3.03 (2.38-3.85)^e^
	Model 3^d^	567 (1.4%)	Ref 1.0	2.01 (1.64-2.45)^e^	2.87 (2.21-3.72)^e^
Myocardial infarction		Events, n (%)^a^	230 (0.6)	109 (1.4)	62 (2.6)
	Model 1^b^	401 (0.8%)	Ref 1.0	1.97 (1.55-2.51)^e^	3.47 (2.56-4.69)^e^
	Model 2^c^	371 (0.8%)	Ref 1.0	1.79 (1.39-2.32)^e^	2.94 (2.13-4.05)^e^
	Model 3^d^	309 (0.8%)	Ref 1.0	1.88 (1.43-2.48)^e^	2.72 (1.90-3.89)^e^
Stroke		Events, n (%)^a^	292 (0.7)	108 (1.3)	57 (2.4)
	Model 1^b^	457 (0.9%)	Ref 1.0	1.35 (1.07-1.70)^e^	2.09 (1.55-2.82)^e^
	Model 2^c^	431 (0.9%)	Ref 1.0	1.35 (1.07-1.72)^e^	1.92 (1.40-2.63)^e^
	Model 3^d^	374 (0.9%)	Ref 1.0	1.25 (0.97-1.62)	1.93 (1.38-2.70)^e^

HRs, 95% CI for atherosclerotic cardiovascular disease, coronary artery disease, myocardial infarction, and stroke according to extent of machine learning-derived abdominal aortic calcification scores (ML-AAC24).

ML-AAC24 = machine learning-derived abdominal aortic calcification 24-point algorithm.

a Number of events across the extent of ML-AAC24 categories are based on the sample size as part of model 1.

b Adjusted for age and sex, n = 50,923.

c Model 2 adjusted for model 1 + body mass index (BMI), systolic blood pressure, physical activity, smoking, prevalent diabetes, country of residence, year imaging was obtained, and ethnicity, n = 47,148.

d Model 3 adjusted for the same covariates as model 2 but with the removal of BMI and the inclusion of total cholesterol and high-density lipoprotein instead, n = 41,175.

e Significantly different (*P* < 0.05) than low ML-AAC24.

When considering the gradient of risk by ML-AAC24 deciles, there was increasing ASCVD risk for ML-AAC24 deciles 8, 9, and 10, compared to the combined referent deciles 1 to 7 (Supplemental Figure 6). Finally, when we removed the 497 individuals with expert AAC scores used for fine-tuning and re-ran the prospective analysis with ASCVD, results remained unchanged. For example, in the multivariable-adjusted analysis (model 3), compared to low ML-AAC24, those with moderate (HR: 1.67 95% CI: 1.43-1.96 vs HR: 1.66, 95% CI: 1.42-1.95) and high ML-AAC24 (HR: 2.49, 95% CI: 2.02-3.06 vs HR: 2.46, 95% CI: 2.00-3.02) had greater ASCVD risk.

Exploratory analysis of the relative importance of all predictors (model 3), when considering the relationship between extent of ML-AAC24 and ASCVD, is presented in Supplemental Figure 7. The largest change in log-likelihood chi-square with the removal of the variable from model 3 was recorded for age (chi-square = 91.4), extent of ML-AAC24 (chi-square = 69.0), sex (chi-square = 34.3), SBP (chi-square = 31.8), and HDL (chi-square = 22.6), indicating they were the top 5 predictors.

### ML-AAC24 with components of incident ASCVD events (CAD, MI, and stroke)

Over a median [IQR] follow-up of 4.1 [3.0-5.6] years, 208,205 total person-years, model 1), 714 (1.4%) of 50,923 participants experienced a CAD event; 401 (0.8%) with MI, and 457 (0.9%) with stroke (Table 2). Increasing extent of ML-AAC24 was associated with stepwise increases in proportions of people experiencing CAD, MI, and stroke events (Figure 1). Compared to low ML-AAC24, moderate and high ML-AAC24 were associated with higher HRs for CAD, MI, or stroke, independent of standard risk factors (Table 2). Similar results were observed in sex-stratified analysis using model 3, (Supplemental Tables 3 and 4); exceptions were moderate ML-AAC24 with stroke in men and high ML-AAC24 with MI and stroke in women (models 2 and 3).

### Sensitivity analyses

Compared to no ML-AAC24, any ML-AAC24 (≥1), observed in 34% of the prospective cohort, was associated with greater relative hazards for ASCVD (HR 1.53, 95% CI: 1.33-1.76), including its components (CAD, MI, and stroke) (model 3) (Supplemental Table 6). When the primary analysis between ML-AAC24 extent and incident ASCVD was restricted to the 41,175 individuals in model 3, results for model 1 and model 2 remained consistent (Supplemental Table 7) with the main analysis with larger sample sizes (n = 50,923 or n = 47,148) (Table 2). ML-AAC24 was weakly correlated with mean (ρ = 0.23, 95% CI: 0.22-0.24, *P* < 0.001) and maximum CIMT (ρ = 0.22, 95% CI: 0.21-0.23, *P* < 0.001) in individuals without prevalent ASCVD. Individuals with moderate and high ML-AAC24 had higher mean and maximum CIMT before (∼4%-7%) and after multivariable adjustment (∼3%-6%) for standard CVD risk factors (Supplemental Table 8). Results for all other exploratory analysis considering ML-AAC24, CIMT, and ASCVD are presented in the Supplementary Results.

## Discussion

In this large prospective study of middle-aged to older adults, we have externally validated a pretrained algorithm to detect and semiquantify AAC24 on LSIs from DXA machines. We found only 5 people in this population needed to be screened to identify 1 person with moderate or high ML-AAC24. Compared to individuals with low ML-AAC24, those with moderate and high ML-AAC24 would be at a substantially higher relative hazard for an incident (first-ever) ASCVD event in the next 5 years (Central Illustration). ML-AAC24 was also weakly correlated with CIMT, an established measure of generalized atherosclerosis.^24^ This study builds on our previous investigation in a bone density registry of predominantly older women (mean ∼75 years, n = 8,565, 6% male), where the extent of ML-AAC24 was also associated with increased risk for major adverse cardiovascular events and death.^13^

**Central Illustration:**
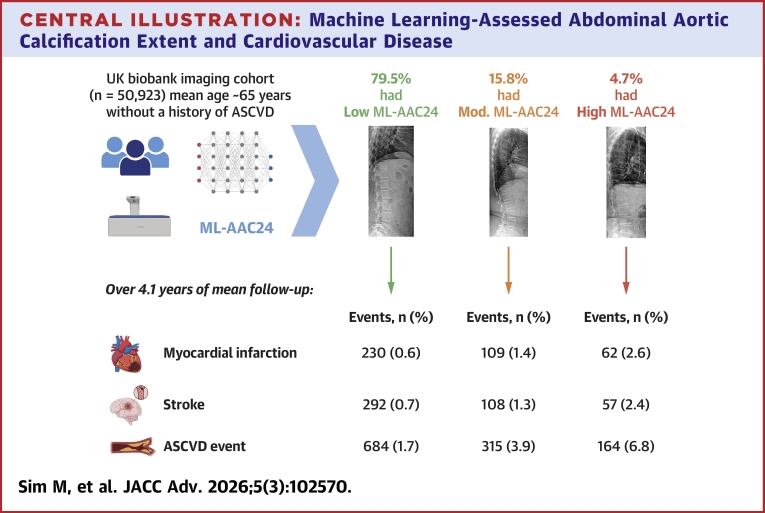
Machine Learning-Assessed Abdominal Aortic Calcification Extent and Cardiovascular Disease Machine learning-derived abdominal aortic calcification scores (ML-AAC24) were assessed from lateral spine images in UK Biobank participants without prior ASCVD. Participants were classified based on extent of ML-AAC24: low (<2), moderate (2 to <6), and high (≥6). Compared to low ML-AAC24, moderate and high ML-AAC24 were associated with progressively increased risks of incident ASCVD, including myocardial infarction and stroke. ASCVD = atherosclerotic cardiovascular disease; ML-AAC24 = machine learning-derived abdominal aortic calcification 24-point algorithm.

While there have been several attempts to automate AAC assessment on bone density machine-derived LSI, this is a challenging task.^11^^,^^12^ Compared to others, we have used the largest expert-annotated training dataset to date. Furthermore, our algorithm is the only one to have been externally validated across different DXA machine manufacturers.^13^ A previous algorithm developed in the UK Biobank used ∼1,000 images with annotations from a subset of the larger cohort (n = 38,264). In conjunction with a lack of adjustment for known CVD risk factors (eg, blood pressure, cholesterol, diabetes, and physical activity), only a modest association with MI was reported (HR: ∼1.4) when comparing people in the highest decile of AAC scores to those in the lowest decile.^11^ We observed a strong and robust gradient of risk for incident ASCVD with increasing ML-AAC24 extent, independent of standard modifiable and nonmodifiable CVD risk factors. Furthermore, ML-AAC24 extent was the second most important contributor (age was the most important) when considering incident ASCVD.

Moderate (3.7%) and high ML-AAC24 (6.8%) was associated with modest gains in absolute risk prediction for ASCVD, in comparison to low ML-AAC24 (1.7%). Despite this, we only observed a small improvement in the AUC (0.009) with the addition of ML-AAC24 extent to model 3. This is perhaps unsurprising given the relatively young age of the cohort, short follow-up duration, as well as traditional CVD risk factors driving subclinical (AAC) and clinical ASCVD. For comparison, in a large individual study meta-analysis, adding 3 biomarkers (high-sensitivity cardiac troponin I, N-terminal pro-B-type natriuretic peptide, and high-sensitivity C-reactive protein) to standard risk factors similarly increased the AUC by 0.009 (95% CI: 0.006-0.012) for ASCVD.^25^ Other work also demonstrates that the inclusion of CAC typically improves the AUC by 0.012 and 0.02 when considering ASCVD events^26^ and CVD deaths,^27^ respectively. Collectively, our findings support ML-AAC24 being a novel and clinically important marker for identifying individuals with high ASCVD risk, as well as correctly reclassifying individuals at low risk.

Measures of subclinical ASCVD, such as CAC imaging, are considered “risk enhancers” to guide treatment decisions.^28^ Similarly, our findings suggest that AAC may also be considered as a “risk enhancer.” This is supported by the favorable test characteristics of ML-AAC24, including rapid (55 DXA LSI images a second) and reproducible scanning using widely available DXA machines with very low radiation exposure (<20 μSv). Indeed, our algorithm can be applied seamlessly to LSIs that are routinely and inexpensively captured using most DXA machines (GE and Hologic) clinically used for vertebral fracture assessment as part of osteoporosis screening. For example, in England, close to 50,000 DXA scans were taken in June 2025.^29^ DXA machines are also increasingly used in younger populations for body composition assessments.^30^ This provides an opportunity for AAC assessment to occur much earlier in life. Notably, the middle-aged cohort representative of the UK Biobank Imaging Study would also serve as an ideal population for ASCVD screening and prevention efforts. Of importance, providing imaging results (eg, carotid and coronary) to individuals presenting with subclinical CVD has been reported to improve risk factor control.^31^^,^^32^ Indeed, we recently completed the first randomized controlled trial demonstrating that the provision of AAC scores with tailored education improved some CVD risk factor management over 12 weeks, compared to those receiving education alone.^33^

Strengths of this study include the use of a pretrained algorithm, with good agreement levels in the subset with expert assessment by a globally recognized expert on AAC assessment from LSI (J.T.S.). Secondly, the large cohort size provides estimates of the prevalence and extent of AAC in this population. Thirdly, we demonstrate a relationship between ML-AAC24 and CIMT supporting its use as a structural marker of subclinical ASCVD. Fourthly, this is the largest study investigating the association between AAC with prevalent and incident ASCVD events. Our study provides robust estimates of the gradient of risk and its prognostic importance in middle-aged to older adults. Finally, this work provides a new cardiovascular “phenotype” for researchers, especially those utilizing the UK Biobank Imaging data to investigate determinants of cardiometabolic health.

### Study Limitations

We note limitations of this work. The UK Biobank Imaging Study is predominantly White, hence the generalizability of our findings to other populations is limited. Further, the cohort may not be representative of the general population, given “healthy volunteer” selection bias.^34^ There was also missing data leading to smaller sample sizes, especially when including biochemistry. This may lead to an underestimate of AAC prevalence in UK adults or similarly aged cohorts. Second, besides CIMT, we did not have other established atherosclerosis measures, particularly of coronary arteries. Nevertheless, AAC has been shown to predict CVD in people with no CAC^6^ and predict CVD independent of CAC.^35^ Third, the median follow-up of ∼4.1 years may not have allowed adequate observation to capture the full risk of ASCVD events, especially in this relatively young cohort. Fourth, the algorithm tended to underestimate AAC24 scores, suggesting further development may be needed. Despite this, ML-AAC24 scores were still strongly predictive of clinical outcomes, and no one identified as high risk was misclassified as low risk. Further, the tendency to underestimate high-range scores as moderate-range scores would still serve as a “risk enhancer” as these individuals would also be considered at elevated risk. Finally, due to the observational nature of this study, causation cannot be established, and residual confounding remains possible.

## Conclusions

Our pretrained algorithm to assess extent of AAC (ML-AAC24) identified individuals with high risk of incident ASCVD events, independent of CVD risk factors. Future studies assessing whether providing these results to people will result in CVD risk-reducing behaviors and management are warranted.

Perspectives **COMPETENCY IN MEDICAL KNOWLEDGE:** Our pretrained algorithm automatically assessed abdominal aortic calcification 24-point scores (ML-AAC24) from lateral spine bone density machine images. In the large observational UK Biobank Imaging Study, moderate to high ML-AAC24 was seen in 1 in 5 people. These individuals also presented with 2 to 3 times greater risk of incident ASCVD events, even after adjusting for traditional risk factors, compared to those with low ML-AAC24.

**TRANSLATIONAL OUTLOOK:** Assessment of ML-AAC24 using widely available bone density machines can identify high-risk individuals with subclinical ASCVD, providing a promising opportunity to alter their trajectory of disease.

## Funding support and author disclosures

The study was supported by a National Health and Medical Research Council (10.13039/501100000925NHMRC) of Australia Ideas grant (APP1183570) and a Medical Research Future Fund 2022 Cardiovascular Health Mission grant (MRF2024225). The salary of Dr Sim is supported by a Royal Perth Hospital Research Foundation Fellowship (RPHRF CAF 00/21) and an Emerging Leader Fellowship from the Western Australian Future Health Research and Innovation Fund. The salary of Dr Smith is supported by a Heart Foundation Postdoctoral Fellowship (Award number: 107194) from the 10.13039/501100001030National Heart Foundation of Australia. The salary of Dr Kemp is funded by an NHMRC of Australia Investigator grant (GNT1177938) and by the 10.13039/501100018897Lions Medical Research Foundation (2020 Lions Dunning-Orlich Investigator Award). Dr Toro-Huamanchumo is supported by the 10.13039/100015742Forrest Research Foundation Scholarship and the 10.13039/501100001798Edith Cowan University (ECU) Higher Degree by Research Scholarship. The salary of Dr Lewis is supported by a National Heart Foundation of Australia Future Leader Fellowship (ID: 102817 & 107323). The salary of Dr Gilani is partly supported by the 10.13039/501100001063Raine Medical Research Foundation through a Raine Priming Grant. The salary of Dr Bondonno is funded by an NHMRC of Australia Early Career Fellowship (Grant number APP1159914). Dr Harvey is supported by the 10.13039/501100000265UK Medical Research Council (MRC) [MC_PC_21003 & 21001] and 10.13039/501100022419NIHR Southampton Biomedical Research Centre, 10.13039/501100000739University of Southampton and 10.13039/100010417University Hospital Southampton NHS Foundation Trust, UK. None of the funding agencies had any role in the conduct of the study; collection, management, analysis, or interpretation of the data; or preparation, review, or approval of the manuscript. The authors have reported that they have no relationships relevant to the contents of this paper to disclose.
