# Supplementary material for: Automated Abdominal Aortic Calcification Scores and Atherosclerotic Cardiovascular Disease in the UK Biobank Imaging Study
Source: JACC Adv. 2026 Jan 29;5(3):102570. doi: 10.1016/j.jacadv.2025.102570 (PMC12874814; doi:10.1016/j.jacadv.2025.102570)
Supplement: Supplemental Methods, Tables and Figures [file mmc1.pdf]

Supplemental Appendix

Supplementary Methods

Machine learning-derived abdominal aortic calcification 24 scores (ML-AAC24)

This process has been detailed previously[1]. In the first stage, we extract the region of interest, i.e., lumbar vertebrae and adjacent regions by cropping upper half of a full view thoracolumbar lateral spine image. This cropped image is then resized to 300 x 300 pixels and rescaled between 0 and 1 intensity range. In the second stage, we extract features from these pre-processed images by fine-tunning the weights of a pre-trained ML-AAC24 model.

We employed EfficientNet-V2S as the baseline encoder for feature extraction. This encoder was pre-trained on multiple large-scale datasets, as described previously[1]. The extracted features were passed to a feed-forward network comprising two fully connected layers with 512 and 128 neurons, respectively, each followed by ReLU activation. A final linear output layer was used to regress the AAC24 scores. The model was fine-tuned in an end-to-end fashion using the Adam optimizer over 75 epochs. To mitigate overfitting, we applied a learning rate scheduler (ReduceLROnPlateau) with a patience of five epochs and implemented early stopping if the validation loss did not improve for 15 consecutive epochs. Model weights were saved at the epoch with the lowest validation loss to ensure optimal generalization. This framework is optimized using mean-squared error (MSE).

$$MSE = \frac{1}{n}\sum_{i=1}^{n}(Y_i - Z_i)^2$$

Here, n is the total number of samples in training batch, whereas Y_i and Z_i are the actual and predicted AAC-24 scores for the ith sample, respectively. As the LSIs were 8-bit compared to the usual 14-bit images that the ML-AAC24 was originally trained on, the algorithm was fine-tuned

on 497 randomly selected LSIs assessed by J.T.S. Fine-tuning was undertaken using 10-fold cross-validation input into the previously developed ML-AAC24 algorithm[1]. Each fold consisted of a training set (402 images), validation set (45 images), and test set (50 images).

Description of covariates

For covariates, we preferentially used the specific variable obtained at Instance 2 (2014+, the closest time to LSI). For missing data at Instance 2, we used covariate data from Instance 1 (Instance 1, 2012-2013) and if missing, used Instance 0 (Instance 0, 2006-2010), with specific detail provided in **Supplementary Table 1**. High-density lipoprotein (HDL) and total cholesterol (assayed using the AU5800 analytical platform (Beckman Coulter California, USA) were only measured at the initial assessment visit (Instance 0, 2006-2010) and first repeat assessment (Instance 1, 2012-2013). Thus, HDL and total cholesterol measurements from Instance 1 were used preferentially; values from Instance 0 were adopted in the event of missing data at Instance 1. Based on previous work[2], presence of diabetes mellitus (type 1 or 2) at the time of imaging was determined using the linked health records (hospital inpatient data) based on ICD-9 and ICD-10 codes (**Supplementary Table 1**). Blood pressure (BP) was obtained after 5 minutes of seated rest and measured on two consecutive measures on the same clinic visit with a 1-minute interval using Omron 705 IT electronic BP monitor (OMRON Healthcare). Only individuals with these two consecutive BP measures were included to enable mean BP across each instance per person to be calculated.

Primary clinical outcome (incident ASCVD)

All ASCVD events were identified by linkage to regional-specific electronic health records since 1980 (including: Hospital Episode Statistics inpatient data in England; Patient Episode Database for Wales; and Scottish Morbidity Records in Scotland). Similarly, dates and

causes of death were obtained from death certificates maintained by the National Health Service Information Centre in England and Wales, and the National Health Service Central Register in Scotland. Linkage procedures are described in detail at http://content.digital.nhs.uk/services. A detailed list of the ICD-9, ICD-10 and OPSC codes used to derive the clinical outcomes are found in **Supplementary Table 1**.

Secondary clinical outcomes (incident ASCVD components)

Based on previous work,[2] CAD was characterised as a combination of MI and/or coronary revascularization procedures. Incident MI was determined by examining hospitalisation records confirming acute MI, its associated acute complications, or death certificates listing ischemic heart disease as the cause of death. The incidence of coronary revascularisation was determined by reviewing hospitalisation records to confirm a coronary revascularisation procedure, such as coronary artery bypass graft surgery or percutaneous angioplasty/stent placement. Similarly, incident ischemic stroke was determined by examining hospitalisation records indicating a diagnosis of acute ischemic stroke, which includes cerebral infarction resulting from thrombosis or cerebral atherosclerosis, or cerebrovascular syndromes, along with their acute complications, or death certificates listing ischemic stroke as the cause of death. The specific ICD-9, ICD-10 or OPSC4 codes used to derive these outcomes are found in **Supplementary Table 1**.

Carotid intima media thickness

Participants were positioned in a supine position with the head rotated at 45°. A 5 to 13 MHz linear array transducer imaged the CIMT of the right common carotid artery at an angle of 120° and 150°, and the left common carotid artery at 210° and 240°.[3] At each angle, the mean CIMT was measured 10 mm proximal to the flow divider at end-diastole using automated edge-

detection software. Sonographers assessed CIMT scan quality against predefined UK Biobank criteria, and a senior radiographer reviewed all scans at regular intervals. Approximately 10% of randomly selected scans and borderline quality scans were externally validated at the Oxford Cardiovascular Clinical Research Facility.

Supplementary sensitivity analysis

Cross-sectional analysis between ML-AAC24, CIMT and prevalent ASCVD

Linear regression was used to test for differences in mean and maximum CIMT (in separate analysis) by extent of ML-AAC24 (low, moderate, high). As CIMT data were not normally distributed, log-transformation was applied, with data presented as estimated marginal means and 95% confidence intervals (95%CI). We also examined if ML-AAC24 extent was associated with prevalent ASCVD. Specifically, logistic regression was used to examine the cross-sectional relationship between ML-AAC24 extent and prevalent ASCVD.

ML-AAC24, CIMT and incident ASCVD

To determine if the relationship between extent of both ML-AAC24 and CIMT with incident ASCVD were independent of each other, further analysis simultaneously including both these measures were undertaken. Here, mean CIMT was categorized as low (below the median), moderate (50-74% percentile) or high (≥75 percentile). As CIMT percentiles were adopted, this analysis was restricted to the 34,555 individuals representing complete cases for all variables included in Model 3. The log-likelihood chi-square (χ^2) statistic that was previously used to evaluate the significance of removing individual predictor variables from Model 3 when considering the relationship between ML-AAC24 extent and ASCVD was repeated, but with the addition of CIMT extent (low, moderate and high).

Supplementary Results

ML-AAC24 and CIMT

From the imaging cohort, 42,795 participants without prevalent ASCVD also had both ML-AAC24 and CIMT assessed. This sample size decreased to 39,570 and 34,564 depending on covariate availability (**Supplementary Figure 3**). When considering deciles of ML-AAC24, CIMT increased progressively with increasing ML-AAC24 scores of ≥1 in deciles 8, 9 and 10, when compared with participants in deciles ≤7 (**Supplementary Figure 8**).

ML-AAC24 with prevalent ASCVD

Prevalent ASCVD at imaging was observed in 4.2% overall (2.7% in low, 7.5% in moderate and 16.4% in high ML-AAC24 categories). After adjusting for age and sex, odds of having prevalent ASCVD was higher in those with moderate (OR 1.98, 95%CI [1.78-2.20]) and high ML-AAC24 (OR 3.98, 95%CI [3.51-4.52]), compared to low ML-AAC24. Results remained similar after adjustment for standard CVD risk factors (**Supplementary Table 9**).

ML-AAC24, CIMT and incident ASCVD

The addition of CIMT extent (low, moderate and high) did not alter the association between moderate and high ML-AAC24 with ASCVD, CAD and MI, compared to low ML-AAC24 (**Supplementary Table 10**); for stroke, only high ML-AAC24 was associated with greater hazards. Alternatively, independent of ML-AAC24 extent, only high CIMT when compared to low CIMT was associated with higher hazards of incident ASCVD, CAD and stroke (**Supplementary Table 11**). Exploratory analysis of the relative importance of all predictors in Model 3, including ML-AAC24 and CIMT extent for incident ASCVD (**Supplementary Figure 9**), found the largest increase in log-likelihood χ^2 was recorded for the extent of ML-AAC24 (χ^2=63.3), age (χ^2=62.8), sex (χ^2=29.0), CIMT (χ^2=26.1) and SBP (χ^2=24.0).

Supplementary references

1. Sharif N, Gilani SZ, Suter D, et al. Machine learning for abdominal aortic calcification assessment from bone density machine-derived lateral spine images. *EBioMedicine*. Aug 2023;94:104676.
2. Patel AP, Wang M, Kartoun U, Ng K, Khera AV. Quantifying and understanding the higher risk of atherosclerotic cardiovascular disease among South Asian individuals: results from the UK Biobank prospective cohort study. *Circulation*. 2021;144(6):410-422.
3. Pillay P, Carter J, Taylor H, Lewington S, Clarke R. Independent Relevance of Different Measures of Adiposity for Carotid Intima-Media Thickness in 40 000 Adults in UK Biobank. *Journal of the American Heart Association*. 2023;12(2):e026694.

Supplementary Table 1. UK Biobank field IDs used to extract data for this study.

Field	Field ID	Instance	Notes
Age at imaging	21003	2	-
Body mass index	21001	2, 1,0	Instance 2 data preferably used. If data was still missing, this was replaced with instance 1 followed by instance 0.
Ethnicity	21000	2, 1, 0	Instance 2 data preferably used. If data was missing, this was replaced with instance 1 followed by instance 0.
Systolic blood pressure	4080	2, 1, 0	Instance 2 data preferably used. If data was missing, this was replaced with instance 1 followed by instance 0.
IPAQ	22032	2, 1, 0	Instance 2 data preferably used. If data was missing, this was replaced with instance 1 followed by instance 0.
Smoking status	20116	2, 1, 0	Instance 2 data preferably used. If data was missing, this was replaced with instance 1 followed by instance 0.
Prevalent diabetes	-	-	*ICD-9:* 2500, 25000, 25001, 25009, 25011, 25019, 2503, 2504, 2505, 25099<br><br>*ICD-10:* E10, E10.1, E10.2, E10.3, E10.4, E10.5, E10.6, E10.7, E10.8, E10.9, E11, E11.0, E11.1, E11.2, E11.3, E11.4, E11.5, E11.6, E11.7, E11.8, E11.9, E12, E12.1, E12.8, E12.9, E13, E13.1, E13.2, E13.3, E13.5, E13.6, E13.7, E13.8, E13.9, E14, E14.1, E14.2, E14.3, E14.4, E14.5, E14.6, E14.7, E14.8, E14.9
Region	54	2,1,0	Regions collapsed into England, Scotland and Wales
Year of imaging	53	2	Derived from clinic visit date
Cholesterol-lowering medication	6153, 6177	2, 1, 0	Instance 2 data preferably used. If data was still missing, this was replaced with instance 1 followed by instance 0.
Blood pressure medications	6153, 6177	2, 1, 0	Instance 2 data preferably used. If data was still missing, this was replaced with instance 1 followed by instance 0.
Max CIMT	22672, 22675, 22678, 22681	2	-

Mean CIMT	22671, 22674, 22677, 22680	2	-
High density lipoprotein	30760	1, 0	Instance 1 data preferably used. If data was missing, this was replaced with instance 0.
Total cholesterol	30690	1, 0	Instance 1 data preferably used. If data was missing, this was replaced with instance 0.
**Clinical endpoints**			
Atherosclerotic cardiovascular disease	-	-	*ICD9:*410,4109,411,4119,412,4129,433,434,4331,4339,4349,4359,4369,4370, 4371, 4378, 4379, 4389<br><br>*ICD10:*G45,G45.0,G45.1,G45.3,G45.4,G45.8,G45.9,G46,G46.3,G46.4,G46.5,G46.7,G46.8,I63,I63.0,I63.2,I63.3,I63.5,I63.8,I63.9,I64,I65,I65.0,I65.1,I65.2,I65.3,I65.8,I65.9,I66,I66.0,I66.1,I66.2,I66.3,I66.4,I66.8,I66.9,I67.2,I69.4,I21,I21.0,I21.1,I21.2,I21.3,I21.4,I21.9,I22,I22.0,I22.1,I22.8,I22.9,I23,I23.0,I23.1,I23.2,I23.3,I23.4,I23.5,I23.6,I23.8,I24,I24.0,I24.1,I24.8,I24.9,I25.2<br><br>*OPCS4:* K40,K40.1,K40.2,K40.3,K40.4,K40.8,K40.9,K41,K41.1,K41.2,K41.3,K41.4,K41.8,K41.9,K42,K42.1,K42.2,K42.3,K42.4,K42.8,K42.9,K43,K43.1,K43.2,K43.3,K43.4,K43.8,K43.9,K44,K44.1,K44.2,K44.8,K44.9,K45.1,K45.2,K45.3,K45.4,K45.5,K45.6,K45.8,K45.9,K46,K46.1,K46.2,K46.3,K46.4,K46.5,K46.8,K46.9,K49.1,K49.2,K49.3,K49.4,K49.8,K49.9,K50.1,K50.2,K50.4,K75.1, K75.2,K75.3,K75.4,K75.8,K75.9
Coronary artery disease	-	-	*ICD-9*: 410,4109,411,4119,412,4129<br><br>*ICD-10*: I21,I21.0,I21.1,I21.2,I21.3,I21.4,I21.9,I22,I22.0,I22.1,I22.8,I22.9,I23,I23.0, I23.1,I23.2,I23.3,I23.4,I23.5,I23.6,I23.8,I24,I24.0,I24.1,I24.8,I24.9,I25.2<br><br>*OPCS4:* K40,K40.1,K40.2,K40.3,K40.4,K40.8,K40.9,K41,K41.1,K41.2,K41.3,K41.4,K41.8,K41.9,K42,K42.1,K42.2,K42.3,K42.4,K42.8,K42.9,K43,K43.1,K43.2,K43.3,K43.4,K43.8,K43.9,K44,K44.1,K44.2,K44.8,K44.9,K45.1,K45.2,K45.3,K45.4,K45.5,K45.6,K45.8,K45.9,K46,K46.1,K46.2,K46.3,K46.4,K46.5,K46.8,K46.9,K49.1,K49.2,K49.3,K49.4,K49.8,K49.9,K50.1,K50.2,K50.4,K75.1,K75.2,K75.3,K75.4,K75.8,K75.9
Myocardial infarction	-	-	*ICD-9:* 410,4109,411,4119,412,4129

			ICD-10: I21,I21.0,I21.1,I21.2,I21.3,I21.4,I21.9,I22,I22.0,I22.1,I22.8,I22.9,I23,I23.0,I23.1,I23.2,I23.3,I23.4,I23.5,I23.6,I23.8
Ischemic stroke	-	-	*ICD-9:*433,434,4331,4339,4349,4359,4369,4370,4371,4378,4379,4389<br><br>*ICD-10:*G45,G45.0,G45.1,G45.3,G45.4,G45.8,G45.9,G46,G46.3,G46.4,G46.5,G46.7,G46.8,I63,I63.0,I63.2,I63.3,I63.5,I63.8,I63.9,I64,I65,I65.0,I65.1,I65.2,I65.3,I65.8,I65.9,I66,I66.0,I66.1,I66.2,I66.3,I66.4,I66.8,I66.9,I67.2,I69.4

Supplementary Table 2. Performance of the machine-learning algorithm assessing abdominal aortic calcification 24 scores (ML-AAC24) compared to expert AAC24 scores for the classification of AAC extent in 497 images.

	**Low (n=343)**	**Moderate (n=121)**	**High (n=33)**	**Average**
Accuracy (%)	84.9	81.9	97.0	87.9
Sensitivity (%)	95.9	47.1	63.6	68.8
Specificity (%)	60.4	93.1	99.4	84.3
Positive Predictive Value (%)	84.4	68.7	87.5	80.2
Negative Predictive Value (%)	86.9	84.5	97.5	89.6

Low: (AAC24 <2), Moderate: (AAC24 2 to <6), High: (AAC24 ≥6).

Supplementary Table 3. Hazard ratios (HR, 95% confidence interval) for the relationship between the severity of machine learning derived abdominal aortic calcification scores (ML-AAC24) with incident atherosclerotic cardiovascular disease (ASCVD), coronary artery disease (CAD), myocardial infarction (MI) and stroke in men.

			**Low ML-AAC24 HR (95%CI)**	**Moderate ML-AAC24 HR (95%CI)**	**High ML-AAC24 HR (95%CI)**
**Atherosclerotic cardiovascular disease**		Events, n (%)[a]	453 (2.5)	209 (5.0)	129 (9.3)
	Model 1[b]	791 (3.3)	Ref 1.0	1.69 (1.43-2.01)*	3.07 (2.49-3.79)*
	Model 2[c]	747 (3.3)	Ref 1.0	1.64 (1.38-1.97)*	2.72 (2.18-3.39)*
	Model 3[d]	634 (3.2)	Ref 1.0	1.58 (1.30-1.92)*	2.63 (2.07-3.34)*
**Coronary artery disease**		Events, n (%)[a]	284 (1.6)	153 (3.6)	91 (6.6)
	Model 1[b]	528 (2.2)	Ref 1.0	2.10 (1.70-2.59)*	3.73 (2.89-4.81)*
	Model 2[c]	495 (2.2)	Ref 1.0	2.07 (1.66-2.57)*	3.36 (2.56-4.40)*
	Model 3[d]	417 (2.1)	Ref 1.0	1.98 (1.56-2.51)*	3.11 (2.32-4.18)*
**Myocardial infarction**		Events, n (%)[a]	166 (0.9)	75 (1.8)	51 (3.7)
	Model 1[b]	292 (1.2)	Ref 1.0	1.85 (1.39-2.48)*	3.83 (2.72-5.39)*
	Model 2[c]	273 (1.2)	Ref 1.0	1.74 (1.29-2.36)*	3.35 (2.34-4.80)*
	Model 3[d]	226 (1.1)	Ref 1.0	1.78 (1.29-2.47)*	2.99 (2.00-4.49)*
**Stroke**		Events, n (%)[a]	168 (0.9)	56 (1.3)	42 (3.0)
	Model 1[b]	266 (1.1)	Ref 1.0	1.07 (0.78-1.47)	2.22 (1.55-3.17)*
	Model 2[c]	256 (1.1)	Ref 1.0	1.06 (0.77-1.46)	1.94 (1.33-2.83)*
	Model 3[d]	220 (1.1)	Ref 1.0	1.02 (0.72-1.44)	1.98 (1.32-2.96)*

[a]number of events (%) for the severity of AAC presented are based on the sample size for [b]Model 1 which is adjusted for age, n=23,933. [c]Model 2 adjusted for Model 1 + body mass index (BMI), systolic blood pressure, physical activity, smoking, prevalent diabetes, country of residence and year AAC was obtained and ethnicity, n=22,511. [d]Model 3 adjusted for the same covariates as Model 2 but with the removal of BMI and the inclusion of total cholesterol and high-density lipoprotein instead, n=19,845. *Significantly different ($p<0.05$) than low ML-AAC24.

Supplementary Table 4. Hazard ratios (HR, 95% confidence interval) for the relationship between the severity of machine learning derived abdominal aortic calcification scores (ML-AAC24) with incident atherosclerotic cardiovascular disease (ASCVD), coronary artery disease (CAD), myocardial infarction (MI) and stroke in women.

			**Low ML-AAC24 HR (95%CI)**	**Moderate ML-AAC24 HR (95%CI)**	**High ML-AAC24 HR (95%CI)**
		Events, n (%)[a]	231 (1.0)	106 (2.8)	35 (3.4)
**Atherosclerotic cardiovascular disease**	Model 1[b]	372 (1.4)	Ref 1.0	2.07 (1.62-2.64)*	2.30 (1.58-3.34)*
	Model 2[c]	342 (1.4)	Ref 1.0	2.02 (1.57-2.61)*	1.94 (1.30-2.90)*
	Model 3[d]	299 (1.4)	Ref 1.0	1.88 (1.42-2.48)*	2.05 (1.35-3.11)*
		Events, n (%)[a]	110 (0.5)	56 (1.5)	20 (2.0)
**Coronary artery disease**	Model 1[b]	186 (0.7)	Ref 1.0	2.26 (1.60-3.18)*	2.71 (1.64-4.49)*
	Model 2[c]	172 (0.7)	Ref 1.0	2.11 (1.48-3.01)*	2.02 (1.17-3.50)*
	Model 3[d]	150 (0.7)	Ref 1.0	2.12 (1.45-3.12)*	2.20 (1.24-3.91)*
		Events, n (%)[a]	63 (0.3)	34 (0.9)	11 (1.1)
**Myocardial infarction**	Model 1[b]	109 (0.4)	Ref 1.0	2.28 (1.47-3.55)*	2.48 (1.27-4.87)*
	Model 2[c]	98 (0.4)	Ref 1.0	1.95 (1.22-3.13)*	1.77 (0.84-3.72)
	Model 3[d]	83 (0.4)	Ref 1.0	2.22 (1.33-3.69)*	1.96 (0.88-4.36)
		Events, n (%)[a]	124 (0.6)	52 (1.4)	15 (1.5)
**Stroke**	Model 1[b]	191 (0.7)	Ref 1.0	1.82 (1.29-2.56)*	1.74 (0.99-2.56)
	Model 2[c]	175 (0.7)	Ref 1.0	1.92 (1.34-2.75)*	1.71 (0.95-3.08)
	Model 3[d]	154 (0.7)	Ref 1.0	1.69 (1.14-2.50)*	1.77 (0.96-3.27)

[a]number of events (%) for the severity of AAC presented are based on the sample size for [b]Model 1 which is adjusted for age, n=26,990. [c]Model 2 adjusted for Model 1 + body mass index (BMI), systolic blood pressure, physical activity, smoking, prevalent diabetes, country of residence and year AAC was obtained and ethnicity, n=24,637. [d]Model 3 adjusted for the same covariates as Model 2 but with the removal of BMI and the inclusion of total cholesterol and high-density lipoprotein instead, n=21,330.*Significantly different ($p<0.05$) than low ML-AAC24.

Supplementary Table 5. Hazard ratios (HR, 95% confidence interval) for atherosclerotic cardiovascular disease according to extent of machine learning derived abdominal aortic calcification scores (ML-AAC24) in the multivariable-adjusted analysis (Model 3) with further adjustment for blood pressure and cholesterol medication in the entire cohort (n=40,951), as well as men (n=19,735) and women (n=21,216) separately.

**Atherosclerotic cardiovascular disease**			**Low ML-AAC24 HR (95%CI)**	**Moderate ML-AAC24 HR (95%CI)**	**High ML-AAC24 HR (95%CI)**
**All**	Model 3 + blood pressure medication + cholesterol medication	Events, n (%)[a]	542 (1.7)	251 (3.9)	130 (6.7)
			Ref 1.0	1.65 (1.41-1.94)[*]	2.32 (1.87-2.86[*]
**Male**		Events, n (%)[a]	356 (2.4)	170 (4.9)	101 (8.8)
			Ref 1.0	1.57 (1.29-1.91) [*]	2.51 (1.96-3.21) [*]
**Female**		Events, n (%)[a]	186 (1.1)	81 (2.8)	29 (3.7)
			Ref 1.0	1.85 (1.40-2.44) [*]	1.92 (1.26-2.93) [*]

Model 3 included age, sex, systolic blood pressure, physical activity, smoking, prevalent diabetes, country of residence, year imaging was obtained and ethnicity, total cholesterol and high-density lipoprotein.

Supplementary Table 6. Hazard ratios (HR, 95% confidence interval) for the relationship for the presence of machine learning derived abdominal aortic calcification (ML-AAC24) with incident atherosclerotic cardiovascular disease, coronary artery disease, myocardial infarction and stroke.

			**No ML-AAC24 HR (95%CI)**	**ML-AAC24 present HR (95%CI)**
**Atherosclerotic cardiovascular disease**		Events (%)[a]	540 (1.6)	623 (3.6)
	Model 1[b]	1163 (2.3%)	Ref 1.0	1.67 (1.48-1.90)*
	Model 2[c]	1089 (2.3%)	Ref 1.0	1.62 (1.42-1.85)*
	Model 3[d]	933 (2.3%)	Ref 1.0	1.53 (1.33-1.76)*
**Coronary artery disease**		Events (%)[a]	313 (0.9)	401 (2.3)
	Model 1[b]	714 (1.4%)	Ref 1.0	1.90 (1.63-2.24)*
	Model 2[c]	667 (1.4%)	Ref 1.0	1.86 (1.57-2.20)*
	Model 3[d]	567 (1.4%)	Ref 1.0	1.77 (1.48-2.13)*
**Myocardial infarction**		Events (%)[a]	182 (0.5)	219 (1.3)
	Model 1[b]	401 (0.8%)	Ref 1.0	1.86 (1.50-2.30)*
	Model 2[c]	371 (0.8%)	Ref 1.0	1.75 (1.40-2.20)*
	Model 3[d]	309 (0.8%)	Ref 1.0	1.79 (1.40-2.29)*
**Stroke**		Events (%)[a]	228 (0.7)	229 (1.3)
	Model 1[b]	457 (0.9%)	Ref 1.0	1.35 (1.11-1.65)*
	Model 2[c]	431 (0.9%)	Ref 1.0	1.32 (1.08-1.62)*
	Model 3[d]	374 (0.9%)	Ref 1.0	1.23 (0.98-1.53)

[a] number of events across the severity of ML-AAC24 categories are based on the sample size as part of Model 1; [b] adjusted for age and sex, n=50,923.[c]Model 2 adjusted for Model 1 + body mass index (BMI), systolic blood pressure, physical activity, smoking, prevalent diabetes, country of residence, the year AAC was obtained and ethnicity, n=47,148. [d] Model 3 adjusted for the same covariates as Model 2 but with the removal of BMI and the inclusion of total cholesterol and high-density lipoprotein instead, n=41,175.*Significantly different ($p<0.05$) than no ML-AAC24.

Supplementary Table 7: Hazard ratios (HR, 95% confidence interval) for incident atherosclerotic cardiovascular disease (ASCVD), coronary artery disease, myocardial infarction and stroke according to machine learning derived abdominal aortic calcification scores (ML-AAC24) in 41,175 individuals, representing the sample size of Model 3 in the primary analysis, across all models of adjustment.

			**Low ML-AAC24 HR (95%CI)**	**Moderate ML-AAC24 HR (95%CI)**	**High ML-AAC24 HR (95%CI)**
**Atherosclerotic cardiovascular disease**		Events, n (%)	548 (1.7)	252 (3.9)	133 (6.8)
	Model 1	933 (2.3%)	Ref 1.0	1.82 (1.55-2.13)*	2.90 (2.37-3.56)*
	Model 2		Ref 1.0	1.71(1.46-2.00)*	2.52 (2.05-3.10)*
	Model 3		Ref 1.0	1.66 (1.42-1.95)*	2.46 (2.00-3.02)*
**Coronary artery disease**		Events, n (%)	310 (1.0)	170 (2.6)	87 (4.5)
	Model 1	567 (1.4%)	Ref 1.0	2.23 (1.83-2.73)*	3.48 (2.69-4.49)*
	Model 2		Ref 1.0	2.08 (1.71-2.55)*	2.99 (2.30-3.88)*
	Model 3		Ref 1.0	2.01 (1.64-2.45)*	2.87 (2.21-3.72)*
**Myocardial infarction**		Events, n (%)	176 (0.5)	88 (1.4)	45 (2.3)
	Model 1	309 (0.8%)	Ref 1.0	2.12 (1.62-2.79)*	3.35 (2.35-4.76)*
	Model 2		Ref 1.0	1.97 (1.49-2.59)*	2.84 (1.99-4.07)*
	Model 3		Ref 1.0	1.88 (1.43-2.48)*	2.72 (1.90-3.89)*
**Stroke**		Events, n (%)	240 (0.7)	86 (1.3)	48 (2.5)
	Model 1	374 (0.9%)	Ref 1.0	1.32 (1.02-1.71)*	2.15 (1.55-2.99)*
	Model 2		Ref 1.0	1.26 (0.97-1.63)	1.94 (1.39-2.71)*
	Model 3		Ref 1.0	1.25 (0.97-1.62)	1.93 (1.38-2.70)*

Model 1 adjusted for age and sex; Model 2 adjusted for Model 1 + body mass index (BMI), systolic blood pressure, physical activity, smoking, prevalent diabetes, country of residence, the year AAC was obtained and ethnicity; and Model 3 adjusted for the same covariates as Model 2 but with the removal of BMI and the inclusion of total cholesterol and high-density lipoprotein instead. *Significantly different ($p<0.05$) than low ML-AAC24.

Supplementary Table 8. Estimated mean (95% confidence interval) mean and maximum carotid intima-media thickness (CIMT) according to the extent of automated abdominal aortic calcification (ML-AAC24).

		Total n	Low ML-AAC24 Mean (95%CI)	Moderate ML-AAC24 Mean (95%CI)	High ML-AAC24 Mean (95%CI)
**Mean CIMT (mm)**	Model 1[a]	42,795	0.680 (0.679-0.681)$^{\$\wedge}$	0.704 (0.702-0.707)$^{*\wedge}$	0.726 (0.721-0.731)$^{*\$}$
	Model 2[b]	39,570	0.680 (0.679-0.682)$^{\$\wedge}$	0.701 (0.698-0.704)$^{*\wedge}$	0.720 (0.715-0.725)$^{*\$}$
	Model 3[c]	34,564	0.681 (0.680-0.682)$^{\$\wedge}$	0.699 (0.696-0.702)$^{*\wedge}$	0.718 (0.712-0.723)$^{*\$}$
**Maximum CIMT (mm)**	Model 1[a]	42,795	0.791 (0.789-0.792)$^{\$\wedge}$	0.820 (0.817-0.824)$^{*\wedge}$	0.848 (0.842-0.854)$^{*\$}$
	Model 2[b]	39,570	0.791 (0.790-0.793)$^{\$\wedge}$	0.817 (0.813-0.820)$^{*\wedge}$	0.841 (0.834-0.847)$^{*\$}$
	Model 3[c]	34,564	0.792 (0.791-0.794)$^{\$\wedge}$	0.814 (0.810-0.817)$^{*\wedge}$	0.838 (0.831-0.844)$^{*\$}$

Model 1 adjusted for age and sex. Model 2: Model 1 + body mass index (BMI), systolic blood pressure, physical activity, smoking, prevalent diabetes, country of residence, year imaging was obtained and ethnicity. Model 3: same covariates as Model 2 but with the removal of BMI and the inclusion of total cholesterol and high-density lipoprotein instead. [a] n= 34,207, 6646 and 1942 for low, moderate and high ML-AAC24, respectively; [b] n= 31,720, 6072 and 1778 for low, moderate and high ML-AAC24, respectively; [c] n= 27,668, 5325 and 1571 for low, moderate and high ML-AAC24, respectively. *Significantly different ($p<0.05$) than low ML-AAC24; $^{\$}$significantly different ($p<0.05$) than moderate ML-AAC24; $^{\wedge}$significantly different ($p<0.05$) than high ML-AAC24.

Supplementary Table 9: Odds ratios (OR, 95% confidence interval) for prevalent atherosclerotic cardiovascular disease (ASCVD) according to machine learning derived abdominal aortic calcification scores (ML-AAC24).

			**Low ML-AAC24 OR (95%CI)**	**Moderate ML-AAC24 OR (95%CI)**	**High ML-AAC24 OR (95%CI)**
		Events, n (%)	1113 (2.7)	661 (7.5)	475 (16.4)
**Prevalent atherosclerotic cardiovascular disease**	Model 1	2249 (4.2)	Ref 1.0	1.98 (1.78-2.20)*	3.98 (3.51-4.52)*
	Model 2	2094 (4.2)	Ref 1.0	1.98 (1.78-2.22)*	3.75 (3.28-4.28)*
	Model 3	1843 (4.3)	Ref 1.0	1.88 (1.67-2.12)*	3.37 (2.91-3.89)*

Model 1 (n=53,599) adjusted for age and sex; Model 2 (n=49,640) adjusted for Model 1 + body mass index (BMI), systolic blood pressure, physical activity, smoking, prevalent diabetes, country of residence, the year AAC was obtained and ethnicity; and Model 3 (n=43,362) adjusted for the same covariates as Model 2 but with the removal of BMI and the inclusion of total cholesterol and high-density lipoprotein instead. Model 2 number of events (%) low n=1046 (2.7), moderate n=610 (7.6) and high ML-AAC24 n=438 (16.4). Model 3 number of events (%) low n=921 (2.7), moderate n=545 (7.7) and high ML-AAC24 n=377 (16.1). *Significantly different ($p<0.05$) than low ML-AAC24.

Supplementary Table 10. Hazard ratios (HR, 95% confidence interval) for atherosclerotic cardiovascular disease (ASCVD), coronary artery disease, myocardial infarction and stroke according to extent of machine learning derived abdominal aortic calcification scores (ML-AAC24) in 34,555 individuals with case-complete data across all three models of adjustment that also included the extent of mean carotid intima-media thickness (CIMT).

			**Low ML-AAC24 HR (95%CI)**	**Moderate ML-AAC24 HR (95%CI)**	**High ML-AAC24 HR (95%CI)**
**Atherosclerotic cardiovascular disease**		Events, n (%)	491 (1.8)	228 (4.3)	114 (7.3)
	Model 1		Ref 1.0	1.77 (1.50-2.09)*	2.66 (2.14-3.31)*
	Model 2	833 (2.4)	Ref 1.0	1.68 (1.42-1.99)*	2.37 (1.90-2.95)*
	Model 3		Ref 1.0	1.64 (1.39-1.94)*	2.30 (1.84-2.87)*
**Coronary artery disease**		Events, n (%)	283 (1.0)	152 (2.9)	75 (4.8)
	Model 1		Ref 1.0	2.11 (1.71-2.61)*	3.16 (2.41-4.16)*
	Model 2	510 (1.5)	Ref 1.0	2.00 (1.62-2.47)*	2.76 (2.09-3.64)*
	Model 3		Ref 1.0	1.92 (1.55-2.37)*	2.64 (2.00-3.48)*
**Myocardial infarction**		Events, n (%)	155 (0.6)	81 (1.5)	36 (2.3)
	Model 1		Ref 1.0	2.16 (1.62-2.88)*	2.96 (2.00-4.36)*
	Model 2	272 (0.8)	Ref 1.0	2.02 (1.52-2.70)*	2.56 (1.73-3.81)*
	Model 3		Ref 1.0	1.93 (1.44-2.57)*	2.44 (1.65-3.62)*
**Stroke**		Events, n (%)	212 (0.8)	80 (1.5)	42 (2.7)
	Model 1		Ref 1.0	1.33 (1.01-1.74)*	2.02 (1.43-2.87)*
	Model 2	334 (0.9)	Ref 1.0	1.29 (0.98-1.69)	1.89 (1.33-2.70)*
	Model 3		Ref 1.0	1.28 (0.98-1.68)	1.88 (1.32-2.69)*

Model 1; adjusted for age, sex and mean CIMT extent (low, moderate and high). Model 2 adjusted for Model 1 + body mass index (BMI), systolic blood pressure, physical activity, smoking, prevalent diabetes, country of residence, the year imaging was obtained and ethnicity; and Model 3 adjusted for the same covariates as Model 2 but with the removal of BMI and the inclusion of total cholesterol and high-density lipoprotein instead. *Significantly different ($p<0.05$) than low ML-AAC24.

Supplementary Table 11. Hazard ratios (HR, 95% confidence interval) for atherosclerotic cardiovascular disease (ASCVD), coronary artery disease, myocardial infarction and stroke according to extent of mean carotid intima-media thickness (CIMT) in 34,555 individuals with case-complete data across all three models of adjustment that also included the extent of machine learning derived abdominal aortic calcification scores (ML-AAC24).

			**Low CIMT HR (95%CI)**	**Moderate CIMT HR (95%CI)**	**High CIMT HR (95%CI)**
		Events, n (%)	267 (1.5)	204 (2.4)	362 (4.2)
**Atherosclerotic cardiovascular disease**	Model 1		Ref 1.0	1.19 (0.99-1.44)	1.70 (1.43-2.02)*
	Model 2	833 (2.4)	Ref 1.0	1.14 (0.95-1.37)	1.56 (1.31-1.86)*
	Model 3		Ref 1.0	1.14 (0.94-1.37)	1.55 (1.30-1.84)*
		Events, n (%)	164 (1.0)	128 (1.5)	218 (2.5)
**Coronary artery disease**	Model 1		Ref 1.0	1.20 (0.95-1.53)	1.58 (1.27-1.97)*
	Model 2	510 (1.5)	Ref 1.0	1.14 (0.90-1.44)	1.43 (1.14-1.78)*
	Model 3		Ref 1.0	1.13 (0.89-1.44)	1.41 (1.13-1.76)*
		Events, n (%)	96 (0.6)	66 (0.8)	110 (1.3)
**Myocardial infarction**	Model 1		Ref 1.0	1.09 (0.79-1.51)	1.42 (1.05-1.91)*
	Model 2	272 (0.8)	Ref 1.0	1.02 (0.74-1.40)	1.24(0.92-1.67)
	Model 3		Ref 1.0	1.01 (0.73-1.39)	1.20 (0.89-1.63)
		Events, n (%)	104 (0.6)	80 (0.9)	150 (1.7)
**Stroke**	Model 1		Ref 1.0	1.19 (0.88-1.60)	1.87 (1.42-2.45)*
	Model 2	334 (1.0)	Ref 1.0	1.16 (0.86-1.56)	1.80 (1.36-2.37)*
	Model 3		Ref 1.0	1.16 (0.86-1.57)	1.71 (1.36-2.37)*

Model 1; adjusted for age, sex and ML-AAC24 (low, moderate and high).Model 2 adjusted for Model 1 + body mass index (BMI), systolic blood pressure, physical activity, smoking, prevalent diabetes, country of residence, the year imaging was obtained and ethnicity;and Model 3 adjusted for the same covariates as Model 2 but with the removal of BMI and the inclusion of total cholesterol and high-density lipoprotein instead. *Significantly different ($p<0.05$) than low CIMT.

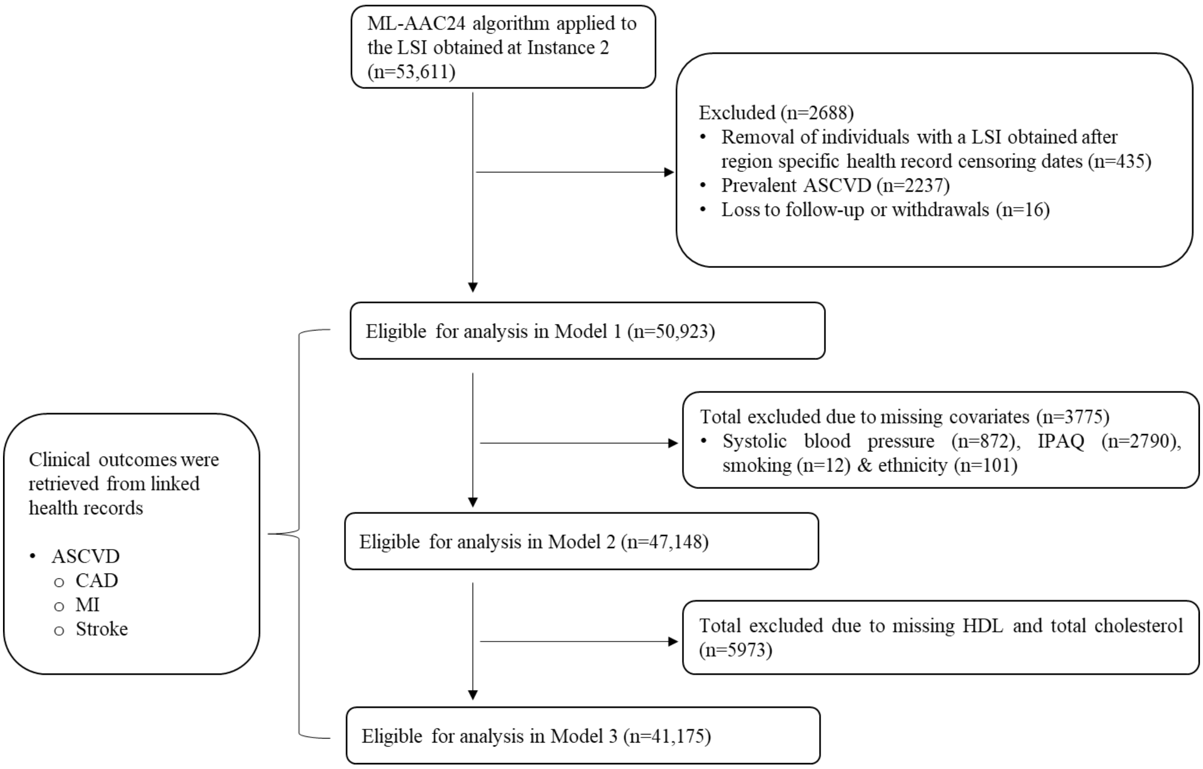

Supplementary Figure 1. Study flowchart for the survival analysis undertaken in the prospective cohort for the relationship between automated abdominal aortic calcification (ML-AAC24) and incident atherosclerotic cardiovascular disease (ASCVD), as well as coronary artery disease (CAD), myocardial infarction (MI) and stroke. LSI, lateral spine image; IPAQ, international physical activity questionnaire (IPAQ); HDL, high density lipoprotein cholesterol (HDL).

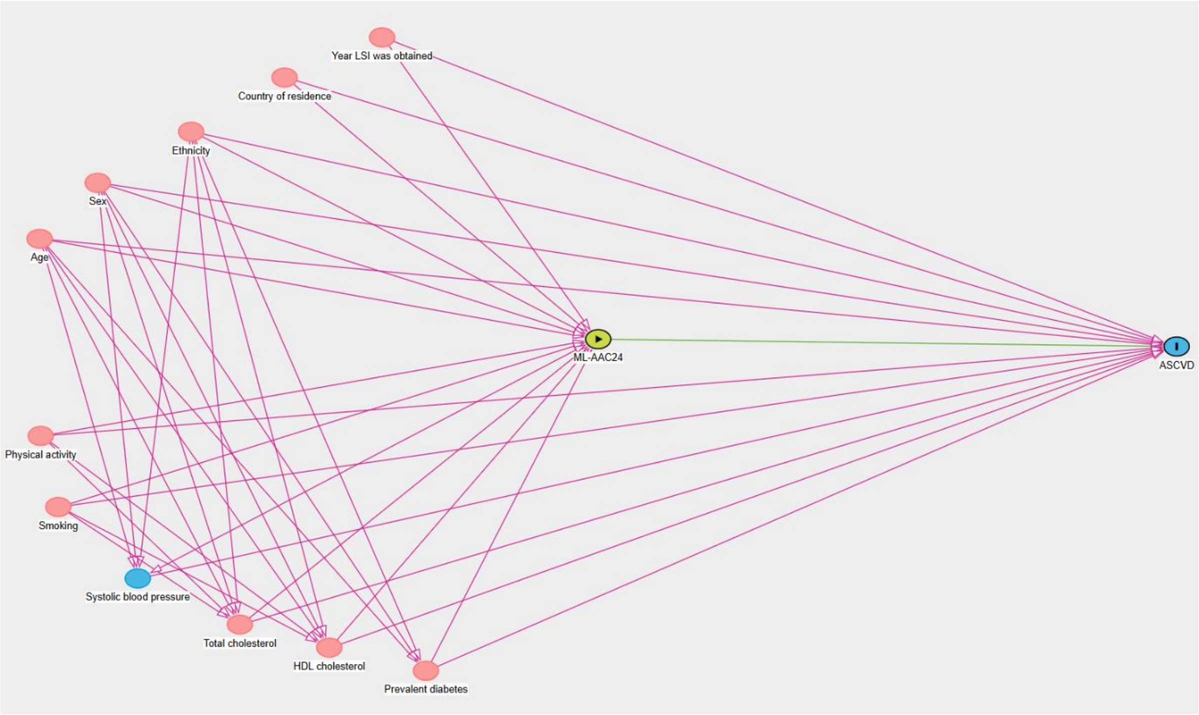

Supplementary Figure 2. Directed acyclic graph (DAG) depicting the hypothesized relationship between machine learning assessed abdominal aortic calcification 24 scores (ML-AAC 24) and incident atherosclerotic cardiovascular disease (ASCVD), with the confounders adjusted for in the analyses (Model 3). LSI, lateral spine image.

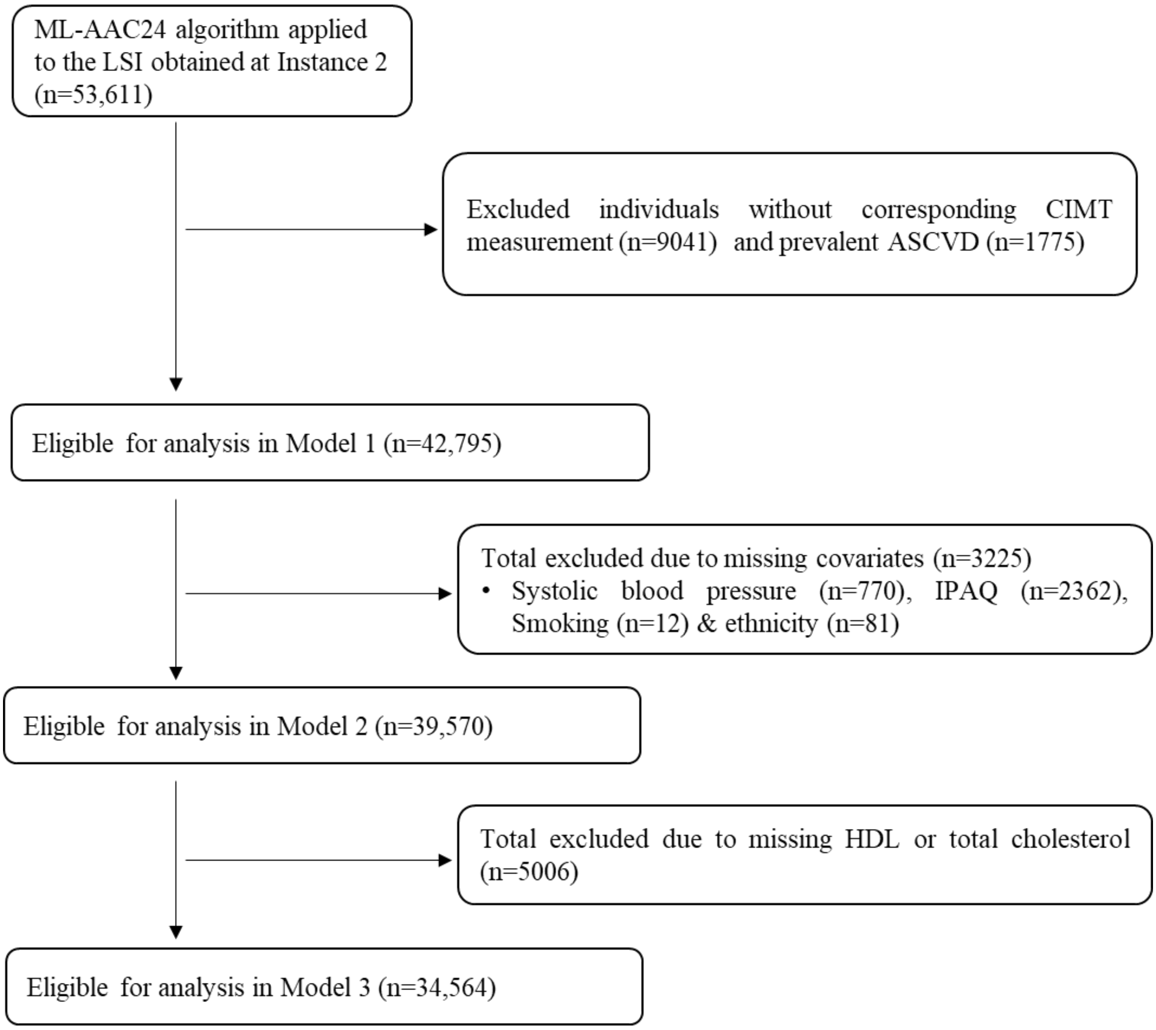

Supplementary Figure 3. Study flowchart for the cross-sectional analysis undertaken in the imaging cohort for the relationship between automated abdominal aortic calcification (ML-AAC24) and carotid intima-media thickness (CIMT). LSI, lateral spine image; IPAQ, international physical activity questionnaire (IPAQ); HDL, high density lipoprotein cholesterol (HDL).

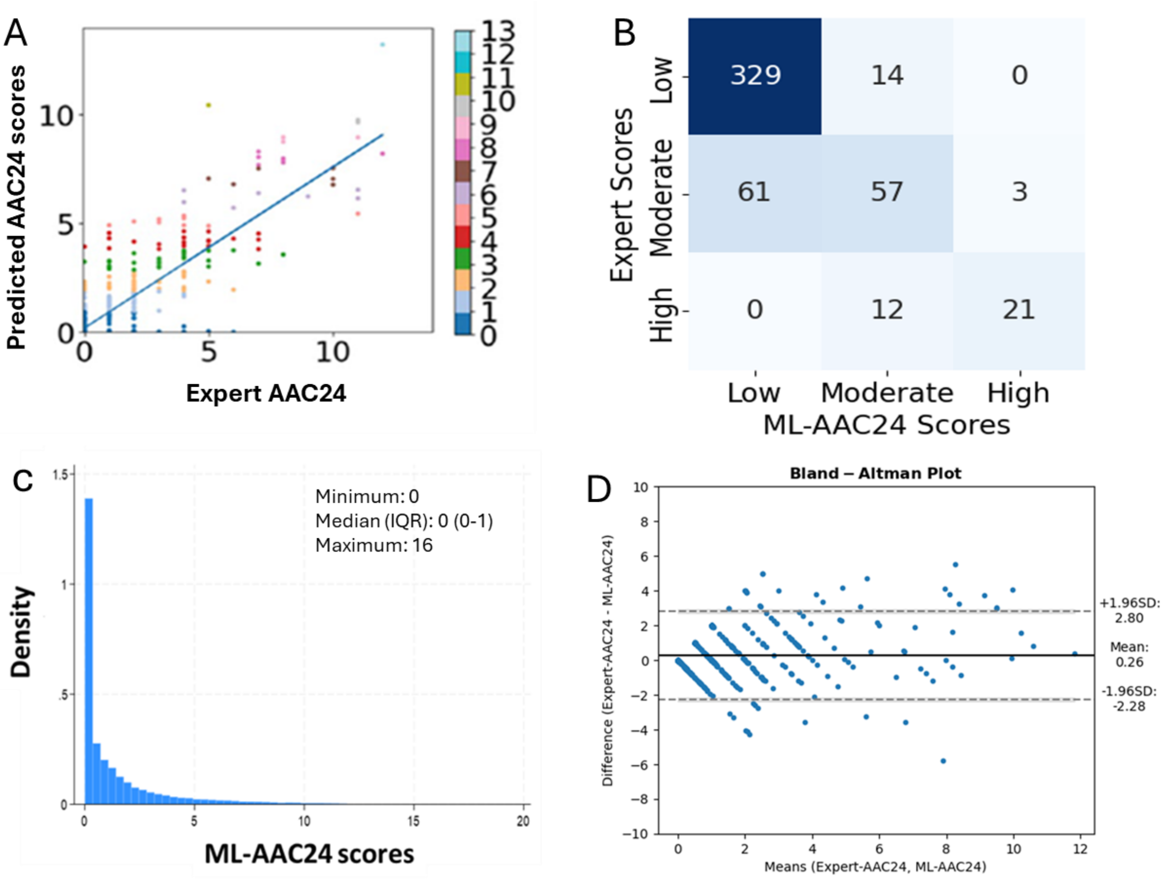

Supplementary Figure 4. Pearsons correlation between expert assessment of AAC24 and ML-AAC24 (r=0.832) in the 497 lateral spine images; (B) Confusion matrix for predicted and expert assessment for group classification (ICC 0.82, 95%CI [0.79-0.85] weighted Cohen's kappa (linear) κ 0.64, 95%CI [0.56-0.71); (C) Distribution of ML-AAC-24 in the 53,611 individuals with lateral spine images available at instance 2; and (D) Bland–Altman plot assessing the agreement between expert AAC24 and ML-AAC24 scores. For 4(A), the colour scale represents the range of predicted ML-AAC-24.

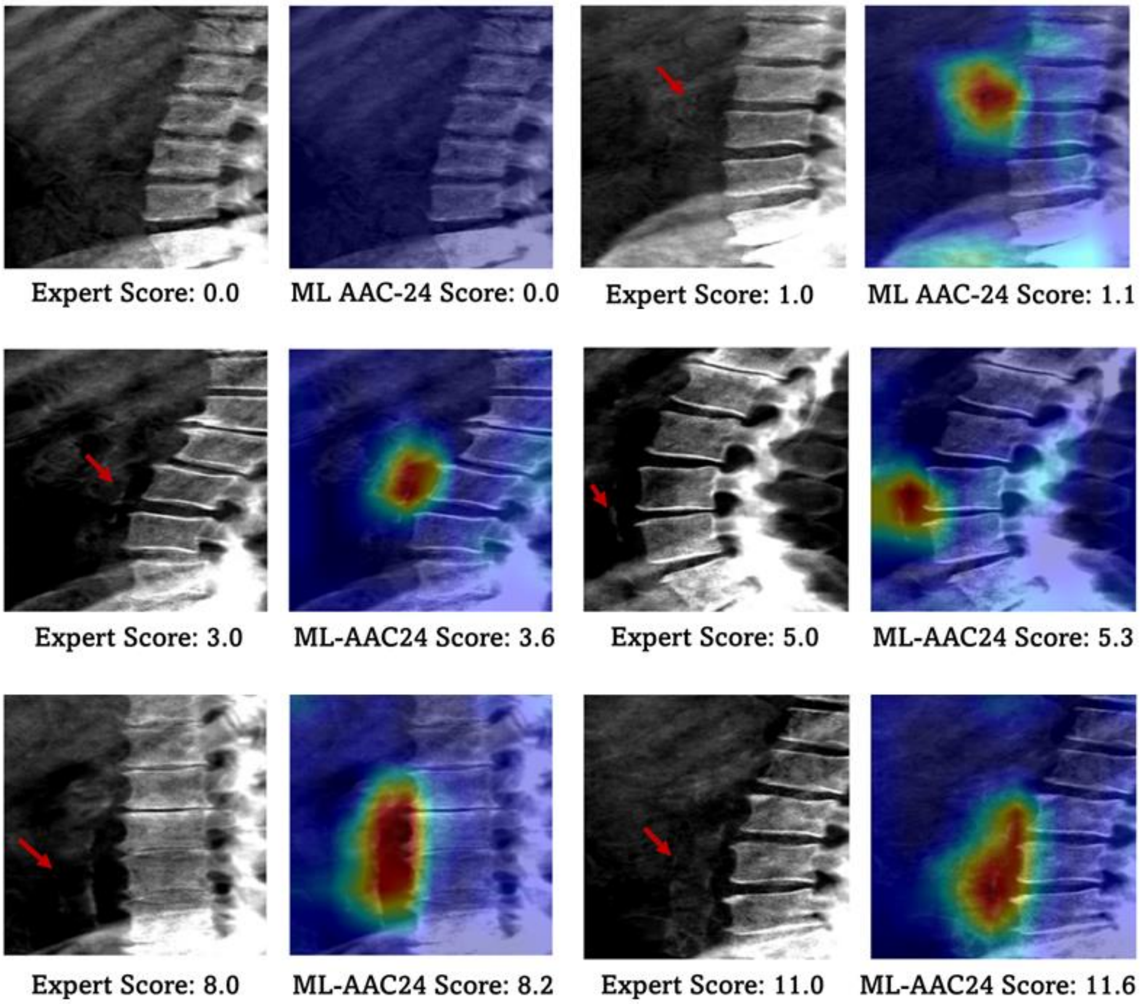

Supplementary Figure 5: Two examples of low (top panels), moderate (middle panels) and high ML-AAC with the qualitative performance of the algorithm on the side-by-side images without (left) and with activation map (right) from the GE iDXA machine lateral spine imaging.

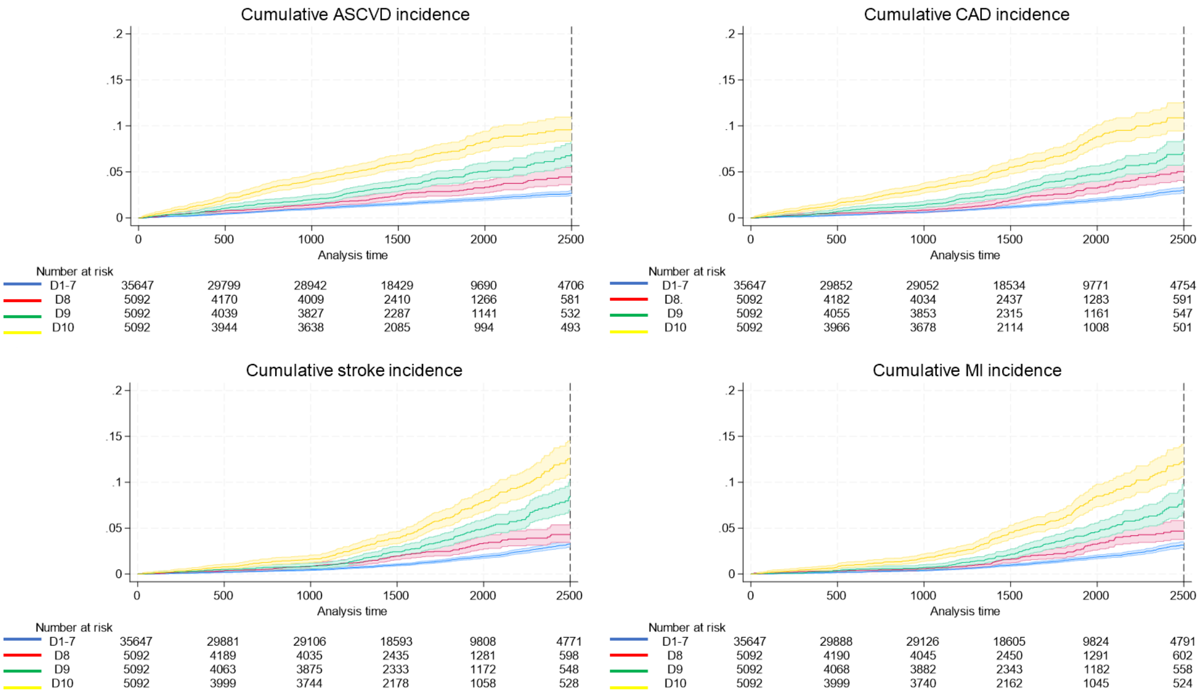

Supplementary Figure 6. Cumulative incidence curves for the relationship between machine learning-derived abdominal aortic calcification deciles with atherosclerotic cardiovascular disease (ASCVD), coronary artery disease (CAD), myocardial infarction (MI) and stroke. Log-rank test $p<0.001$ for all analysis. Deciles 1-7 (blue line & 95%CI, referent), decile 8 (red line), decile 9 (green line), decile 10 (yellow line). X-axis was truncated to 2500 days for visual representation purposes due to low number of individuals with follow-up beyond that time.

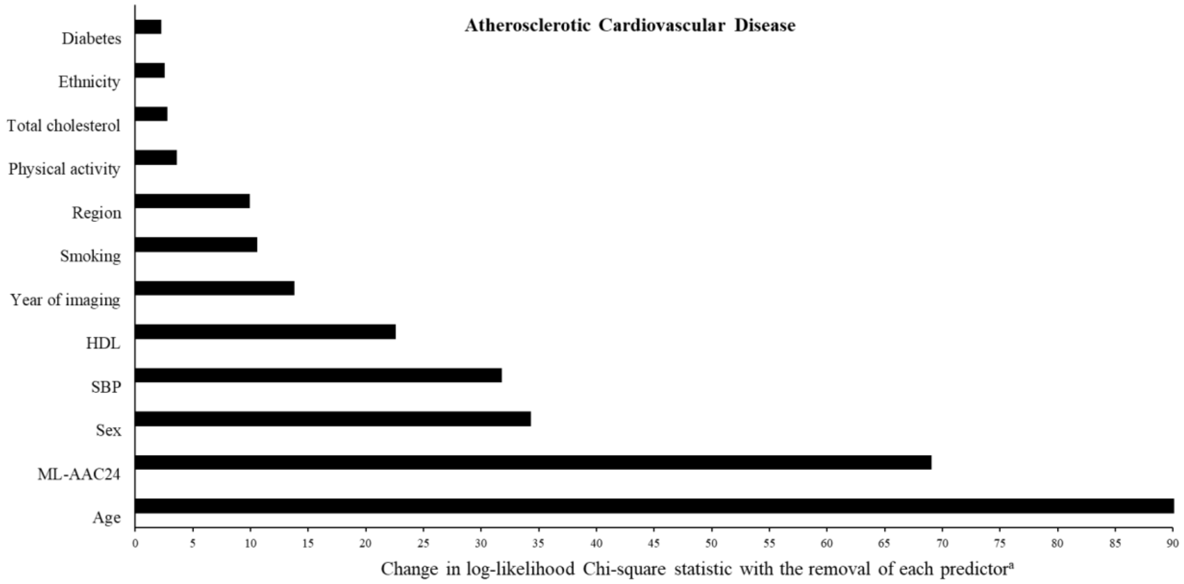

Supplementary Figure 7: Ranking order for the importance of variables in the multivariable-adjusted analysis (Model 3) when considering the risk for incident atherosclerotic cardiovascular disease (ASCVD). Larger log-likelihood Chi-square statistic ($\chi 2$) increases after removal of a predictor from the final model indicates greater importance to the model. SBP, systolic blood pressure; HDL high-density lipoprotein; ML-AAC24, machine learning-derived extent of abdominal aortic calcification.

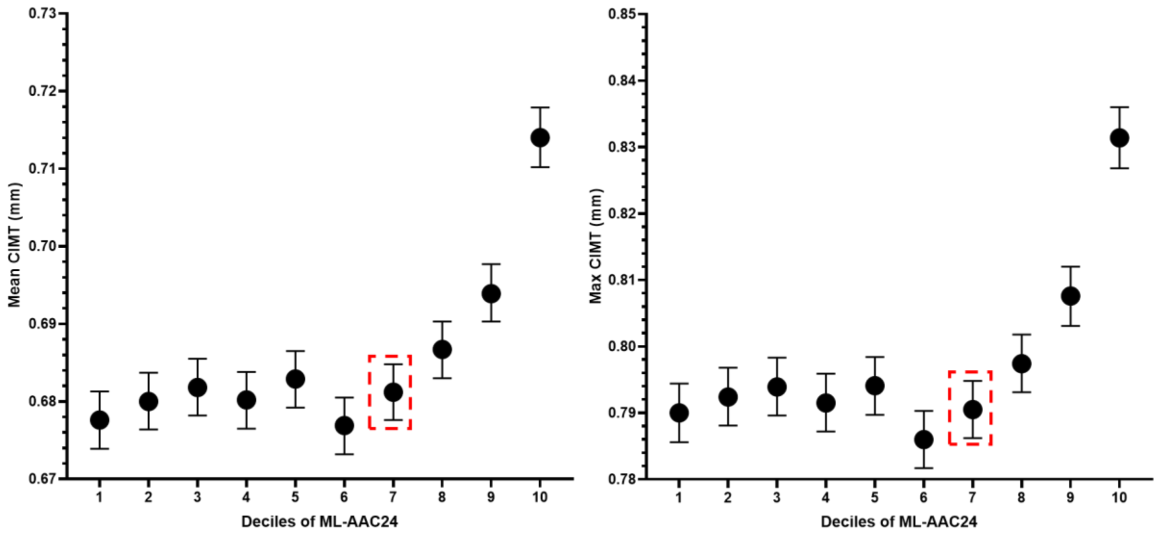

Supplementary Figure 8. Multivariable-adjusted estimated marginal means and 95%CI for (a) mean and (b) maximum carotid intima-media thickness (CIMT) by deciles of machine learning-derived abdominal aortic calcification scores (ML-AAC24). Analysis (Model 3) adjusted for age, sex, systolic blood pressure, physical activity, smoking, prevalent diabetes, country of residence, the year AAC was obtained, ethnicity, total cholesterol and high-density lipoprotein. Red dashed box indicates decile where ML-AAC scores go from absent (<1) to present (≥1).

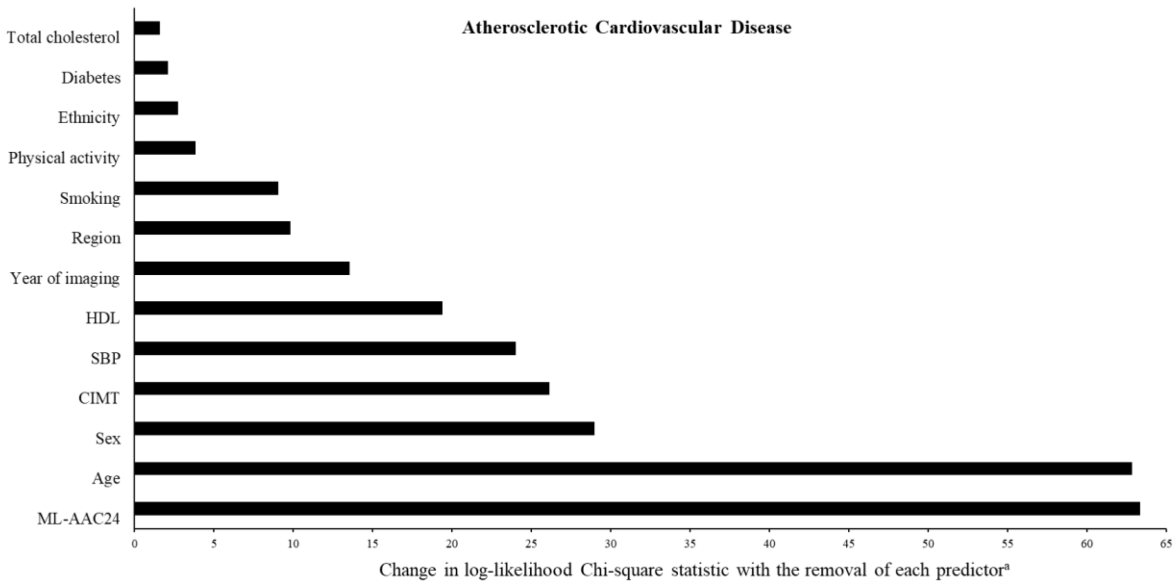

Supplementary Figure 9: Ranking order for the importance of variables in the multivariable-adjusted analysis (Model 3), with the additional inclusion of the extent of mean carotid intima media thickness (CIMT), when considering the risk for incident atherosclerotic cardiovascular disease (ASCVD). Larger log-likelihood Chi-square statistic ($\chi2$) increases after removal of a predictor from the final model indicates greater importance to the model. SBP, systolic blood pressure; HDL high-density lipoprotein; ML-AAC24, machine learning-derived extent of abdominal aortic calcification.
